# Health-related quality of life and its determinants in patients with malignant brain tumors: a prospective longitudinal study

**DOI:** 10.1007/s00520-026-11262-0

**Published:** 2026-09-26

**Authors:** Zhiyuan Xiao, Wenlin Chen, Tianrui Yang, Yaning Wang, Junlin Li, Xiaopeng Guo, Lijun Wang, Dongrui Xu, Wenwen Jiang, Yi Zhang, Yu Wang, Wenbin Ma

**Affiliations:** 1 Department of Neurosurgery, Peking Union Medical College Hospital, Chinese Academy of Medical Sciences and Peking Union Medical College, Beijing, China; 2 Office of Planning and Development, Peking Union Medical College Hospital, Chinese Academy of Medical Sciences and Peking Union Medical College, Beijing, China; 3 Eight-Year Medical Doctor Program, Chinese Academy of Medical Sciences and Peking Union Medical College, Beijing, China; 4 Department of Ultrasound, Dongcheng District, Union Medical College Hospital, Chinese Academy of Medical Sciences and Peking Union Medical College, No.1 Shuaifuyuan, Beijing, 100730 China; 5 Peking Union Medical College Hospital (East), No.1 Shuaifuyuan, Dongcheng District., Beijing, 100730 China

**Keywords:** Malignant brain tumors, HRQoL, Prognostic factors, Assessment, Perioperative

## Abstract

**Objective:**

Evaluate the preoperative health-related quality of life (HRQoL) of patients with malignant brain tumors (MBT) and explore changes in HRQoL and factors associated with it within 3 months after surgery.

**Methods:**

Patients with MBT who underwent surgery were included in the study. Patients were evaluated using the EORTC QLQ-C30 and QLQ-BN20 scales, as well as the Karnofsky Performance Status Scale. Sociodemographic, tumor history, and surgical data were collected.

**Results:**

A total of 140 patients (98 glioma cases and 42 brain metastasis cases) completed the preoperative assessment. Seven days after surgery, overall health status, physical function, role function, fatigue, pain, and lower extremity weakness deteriorated significantly (p < 0.05). However, most glioma patients’ functions returned to baseline levels one month later. Patients with brain metastases experienced a decline in physical, role, and social functions seven days after surgery. One month later, cognitive function, future uncertainty, and headaches improved. Multivariate linear regression analysis revealed that sociodemographic, tumor, and surgical factors significantly predicted various domains of HRQoL at different time points. Overall health condition exhibited a V-shaped trend after the operation.

**Conclusion:**

Age, tumor location, surgical method and KPS score are important factors associated with HRQoL. Regular assessment of these factors may help formulate individualized interventions to optimize patients’ quality of life.

**Supplementary Information:**

The online version contains supplementary material available at https://doi.org/10.1007/s00520-026-11262-0.

## Introduction

Malignant brain tumors include primary intracranial tumors and brain metastases (BM). Glioma is the most common primary malignant tumor of the central nervous system, with an average annual incidence of 4.50 per 100,000 [1]. It has been reported with an approximate incidence rate of 23.12% of gliomas, among malignant cases, glioblastoma multiforme (GBM) was the most common (30.53%), followed by diffuse and anaplastic astrocytoma (25.06%), and oligodendroglioma and oligoastrocytic tumors (14.87%) in the National Brain Tumour Registry of China [2]. Currently, the clinical treatment of glioma includes surgical resection, radiotherapy, chemotherapy, targeted therapy, immunotherapy, and tumor treating fields [3, 4]. In contrast to primary intracranial tumors, brain metastases are common and a serious complication in patients with advanced solid tumors. It is estimated that approximately 10–40% of patients with solid tumors will develop brain metastases, with the majority of brain metastases occurring in patients with lung, breast, colorectal, melanoma or renal cell carcinoma. Multimodal treatments and advances in systemic therapies, which include a combination of surgery, radiotherapy, chemotherapy, immunotherapy and targeted therapies for BM [5–8]. Gliomas and BM portend a poor prognosis. For patients with GBM, median overall survival ranges from 12 to 15 months, and the 5-year survival rate is below 5% [9]. Tumor diagnosis commonly impairs quality of life (QoL), rendering health-related quality of life a critical endpoint alongside survival outcomes [10].

As a patient-reported metric encompassing physical, psychological and social functioning, HRQoL serves as a valuable prognostic indicator linked to symptom burden, treatment toxicity and long-term survival [11, 12]. Prior studies have verified stable QoL following re-irradiation for recurrent glioma and a positive correlation between QoL and psychological resilience [13, 14]. Long-term observations also reveal substantial QoL decline in high-grade glioma (HGG) patients undergoing combined surgical, radiotherapeutic and chemotherapeutic interventions [15]. Accumulated evidence further indicates superior QoL among HGG patients with higher Karnofsky Performance Status (KPS) scores. Existing studies have mainly focused on long-term QoL outcomes after comprehensive anti-tumor therapy, yet few have systematically characterized perioperative HRQoL dynamics in MBT patients within the early postoperative stage. This study addresses this notable research gap to support individualized clinical management. Given comparable surgical stress across glioma and brain metastasis patients undergoing intracranial procedures, we performed a pooled analysis of overall perioperative HRQoL trends, with separate assessments of their unique clinical profiles.

The first three postoperative months constitute a critical period for functional recovery and acute symptom changes in patients with malignant brain tumors. Timely assessment of early postoperative health-related quality of life enables clinicians to provide targeted supportive care and optimize perioperative management, thus carrying important clinical implications. We formulated two primary hypotheses in advance: first, overall HRQoL would decrease sharply in the early postoperative phase and recover gradually thereafter; second, sociodemographic, tumor-related and surgical factors would be independently associated with different HRQoL domains at various follow-up time points. These primary hypotheses were fully pre-specified at the study design stage prior to participant enrollment and data collection. By contrast, analyses of individual questionnaire items, secondary subgroup comparisons and additional correlation analyses were performed as exploratory work with no pre-defined hypotheses.

Surgical resection is the primary treatment for malignant brain tumors. Accordingly, this study aimed to explore perioperative changes in patients’ HRQoL and identify factors associated with postoperative patient-reported outcomes. We included the one-week postoperative assessment, a key time point in routine perioperative evaluation, to capture acute symptoms and immediate functional alterations caused by surgical trauma. This assessment constitutes an indispensable part of comprehensive perioperative HRQoL monitoring.

## Materials and methods

### Study design and eligibility criteria

This study was conducted according to the guidelines laid down in the Declaration of Helsinki and all procedures involving human participants were approved by the Ethics Committee of Peking Union Medical College Hospital before the study was initiated (registration number JS-2012). Written informed consent was obtained from all participants. Patients were eligible if they were aged over 18 years, had a histological diagnosis of malignant brain tumor, and received surgical treatment, preoperative evaluation and 3-month postoperative follow-up at Peking Union Medical College Hospital. Patients with cognitive impairment who could not independently complete the questionnaires were excluded from this study. Patients who refused to participate in the study and perinatal women were also excluded from the study. A non-randomized, prospective, longitudinal study was conducted to assess the scale before and after resection or biopsy surgery, with regular follow-up at 7 days, 1 month, and 3 months after surgery. Participants were contacted by outpatient visit or interval phone calls at each follow-up time point. All eligible patients were consecutively enrolled and data were collected from May 2018 to August 2020.

### Data collection

Trained researchers administered the scales in person during hospitalization and follow-up. For discharged patients who failed to return for scheduled re-examinations, we conducted telephone follow-ups. To guarantee data quality, researchers communicated directly with patients; we only consulted caregivers to verify patients’ general conditions, and no questionnaires were completed by proxy. All scales were independently finished by the patients themselves. We recorded the time to complete each scale, patients’ general conditions, current systemic treatment regimens and hospitalization status. All data were uploaded to an online database.

### Questionnaires and scales

#### General and surgical information

Baseline clinical characteristics were obtained from the individual medical records of enrolled patients, including age, sex, body mass index (BMI), years of education, oncologic history (tumor location, tumor type, tumor grade, histopathology, molecular pathology, and treatment option), radiographic data, and surgical history. Age and education level were recorded to obtain an accurate result. The results of these questionnaires were individually entered into an online database, and each questionnaire contained a subset of independent items.

#### European organization for research and treatment of cancer quality of life questionnaire core 30 (EORTC QLQ-C30 and QLQ-BN20).

The EORTC QLQ questionnaires are the most widely used instruments for assessing both physical and psychological aspects of QoL in cancer patients, with the EORTC QLQ-C30 for general tumor patients and the EORTC QLQ-BN20 for patients with brain neoplasms. The EORTC QLQ-C30 assesses five functional domains (physical, role, emotional, cognitive, social), an overall or global QoL scale, three symptom scales (pain, fatigue, nausea/vomiting), and six individual items (dyspnea, insomnia, loss of appetite, diarrhea, constipation, financial difficulties) [16]. The QLQ-BN20 questionnaire consists of 20 items, most of which are grouped into four functional scales (future uncertainty, visual disturbance, motor dysfunction, communication deficit) and seven individual symptom scores (headache, seizures, drowsiness, hair loss, skin itching, leg weakness, bladder control). The final scores for the scales and items were calculated according to the EORTC QLQ scoring manuals, where the raw scores of one question or the average of 2 to 5 questions are rescaled to a range of 0–100 points. The higher the global health status and functional domain scores, the better. Otherwise, the higher the symptom scores, the worse the performance [17].

For the item assessing hair loss in the QLQ-BN20, all participants were clearly instructed to exclude temporary hair shaving for surgical preparation and only report hair loss resulting from tumor progression or anti-tumor treatments.

### Statistical analysis

Data were analyzed using SPSS Statistics 26.0 (IBM, USA), Prism 8.4.3 (GraphPad Software, USA), and R for graphics. Categorical variables were reported as numbers (percentages), and continuous variables were reported as means (SDs) or medians (with interquartile range [IQR]). Levine's test was used to assess the distribution of data. The unpaired t-test and Mann–Whitney U test were used to compare normally and non-normally distributed variables, respectively. The χ2 test was used to compare categorical variables.

For multivariate linear regression analysis, candidate predictive variables were selected strictly based on published evidence and clinical relevance. We incorporated sociodemographic factors, tumor characteristics and surgical parameters that have been proven to be associated with HRQoL in patients with malignant brain tumors in prior studies. All selected indicators are routinely recorded in clinical work and have practical guiding significance for perioperative evaluation, symptomatic treatment and rehabilitation care. Given the large set of potential variables, we further screened predictors according to their clinical importance, retaining only core indicators with high application value for subsequent regression analysis.

Multivariate linear regression (MLR) with an entry method was used to assess factors independently associated with HRQoL. For multivariate linear regression analyses, we reported unstandardized regression coefficients (B) with standard errors (SE), standardized regression coefficients (β), 95% confidence intervals (95% CIs), t-statistics and corresponding P-values as effect sizes. The overall model fit was evaluated using adjusted R^2^, F-statistic and overall P-value. Variance Inflation Factor (VIF) was calculated to test multicollinearity among independent variables; VIF < 5 indicated no obvious multicollinearity. Pearson correlation was used to analyze the correlation between two variables. Statistical significance was defined as a two-tailed p value of less than 0.05. For patients who dropped out, statistical experts were consulted and multiple imputation was used to reduce selection bias. In this study, domain composite scores of the EORTC QLQ-C30 and QLQ-BN20 were defined as primary outcome measures, while analyses of individual questionnaire items were presented only as secondary exploratory results to avoid excessive multiple comparisons. No pre-study power analysis was conducted. All eligible patients were consecutively enrolled during recruitment, and the sample size was determined based on actual clinical enrollment.

## Result

### Study population

**Fig. 1:**
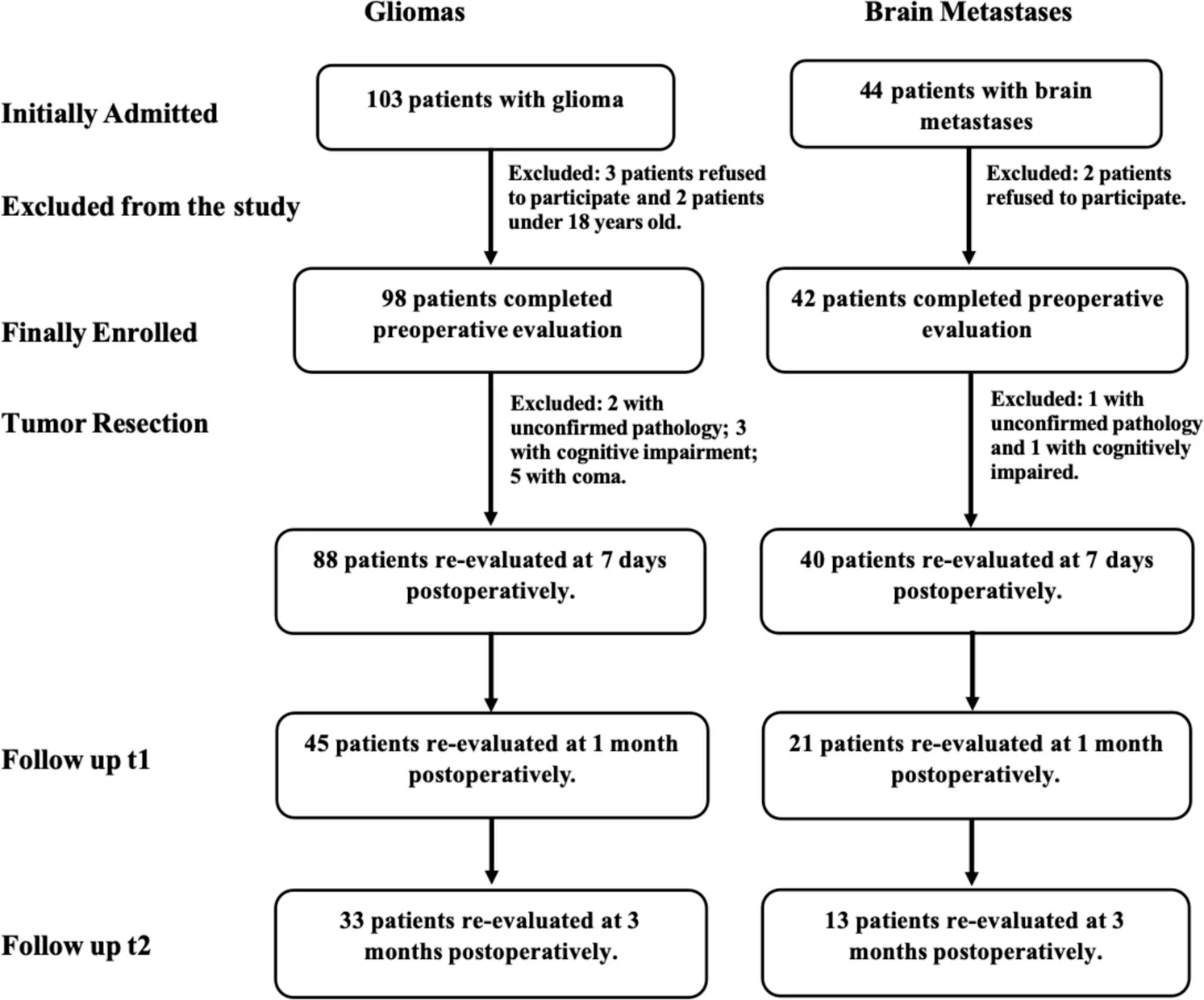
Enrollment and follow-up flow chart

We enrolled 147 patients with malignant brain tumors from May 2018 to August 2020; seven were excluded due to unconfirmed pathology, cognitive impairment or coma. We performed a unified analysis of perioperative HRQoL across all participants, and further conducted subgroup analyses to compare clinical characteristics and HRQoL trajectories between glioma and brain metastases, given their distinct disease features. Ultimately, 140 eligible patients (98 with glioma, 42 with brain metastases) completed preoperative assessments. For the glioma group, 88 (89.8%), 45 (45.9%) and 33 (33.7%) completed the 7-day, 1-month and 3-month postoperative follow-ups respectively. For the BM group, 40 (95.2%), 21 (50%) and 13 (31%) finished assessments at the three time points. The study flow is presented in Fig. 1.

All resected lesions were pathologically confirmed as either glioma or brain metastasis. The cohort included 71 males and 69 females. Glioma patients had a mean age of 47.7 ± 13.9 years, a BMI of 24.4 ± 3.5 kg/m^2^, and 7.4 ± 3.7 years of education; corresponding values for patients with BM were 55.8 ± 12.2 years, 24.0 ± 3.5 kg/m^2^, and 6.3 ± 3.5 years. Most patients with glioma (72/98) underwent gross total resection. Among the 42 patients with bone metastatic tumors, the most common primary tumor was lung cancer (26 cases, 62.0%), followed by breast cancer (8 cases, 19.0%), colorectal carcinoma (3 cases, 7.1%), renal carcinoma (3 cases, 7.1%), and other malignancies (2 cases, 4.8%). Most metastases were located in the right hemisphere.The mean age was 53.1 years for glioblastoma and 55.8 years for BM (*p* = 0.433). Patients with GBM were significantly older than those with other glioma subtypes (*p* = 0.004). Significant intergroup differences were also observed in years of education (*p* = 0.036), tumor hemisphere (*p *= 0.022), tumor location (*p* = 0.018), and surgical approach (*p *= 0.027). Baseline Karnofsky Performance Status (KPS) scores were only available for the glioma group due to incomplete medical records. Detailed demographic and clinical data are summarized in Table 1.

**Table 1:** General information about the enrolled patients with MBT

	Malignant brain tumors (*n* = 140)		t/χ^2^	P
	Gliomas (*n* = 98)	BM (*n* = 42)		
Age, year	47.7 ± 13.9	55.8 ± 12.2	3.669	< 0.001^†^
Age, year (GBM)	53.1 ± 15.2	55.8 ± 12.2	−2.944, #^1^ 0.784, #^2^	0.004^⁋^ 0.433^†^
Sex (no. [%])			2.516	0.113*
Male	54 (55.1)	17 (40.5)		
Female	44 (44.9)	25 (59.5)		
BMI, kg/m^2^	24.4 ± 3.5	24.0 ± 3.5	0.525	0.600^⁋^
Education, year	7.4 ± 3.7	6.3 ± 3.5	−2.099	0.036^†^
Side of the tumor (n. [%])			5.529, λ 5.258, Ө	0.055** 0.022*
Left hemisphere	46 (46.9)	11 (26.2)		
Right hemisphere	50 (51.0)	30 (71.4)		
Bilateral	2 (2.0)	1 (2.4)		
Tumor Location (n. [%])			14.090	0.018**
Frontal lobe	46 (46.9)	17 (40.5)		
Temporal lobe	20 (20.4)	2 (4.8)		
Parietal lobe	10 (10.2)	5 (11.9)		
Occipital lobe	5 (5.1)	6 (14.3)		
Insula lobe	0 (0)	1 (2.4)		
Multiple lobes	15 (15.3)	7 (16.7)		
Other	2 (2.0)	4 (9.5)		
Surgery Option (n. [%])			6.838	0.027**
biopsy	20 (20.4)	2 (4.8)		
subtotal resection	6 (6.1)	1 (2.4)		
gross-total resection	72 (73.5)	39 (92.9)		
Disease Stage (n. [%])				
Newly diagnosis	78 (79.6)	NA		
Recurrence	20 (20.4)	NA		
KPS	86.9 ± 14.4	NA		
Grade (Glioma only) (n. [%])				
1	3 (3.0)	NA		
2	33 (33.6)	NA		
3	28 (28.5%)	NA		
4	34 (34.6)	NA		
Protopathy (BM only) (n. [%])				
Lung cancer	NA	26 (62.0)		
Breast cancer	NA	8 (19.0)		
Colorectal carcinoma	NA	3 (7.1)		
Renal carcinoma	NA	3 (7.1)		
Others^§^	NA	2 (4.8)		

Abbreviation: BM = Brain Metastases; GBM = Glioblastoma Multiforme; BMI = Body mass index; KPS = Karnofsky Performance Status;

⁋ t test.

† Mann‒Whitney U test.

* Chi-square test of Pearson.

** Chi-square exact test of Fisher.

#^1^ Two groups: Age of GBM versus other gliomas. #^2^ Age of GBM versus brain metastatic tumors.

Ө Two groups: left hemisphere versus right hemisphere.

λ Three groups: left hemisphere, right hemisphere and bilateral.

§ Others included frontal sinus squamous cell carcinoma and atrial myxoma.

### Preoperative QLQ-C30 and QLQ-BN20 scores and postoperative changes in patients with glioma.

In terms of domain composite scores, the global health status, physical function and role function of glioma patients decreased significantly at postoperative day 7 (69.1 ± 22.7 vs. 59. 2 ± 25.7, u = −2.541, p = 0.011), (87.4 ± 20.7 vs. 66.4 ± 29.5, u = −5.753, p < 0.001), (79.6 ± 28.6 vs. 60.8 ± 31.1, u = −4.445, p < 0.001), respectively. Meanwhile, obvious changes were observed in overall symptom scores. Additionally, several individual questionnaire items including fatigue, pain, constipation, communication deficit and diarrhea also showed significant deterioration after surgery (*p* < 0.001, *p* = 0.001, *p* = 0.014, *p* = 0.01, respectively). There was a statistically significant difference in the EORTC QLQ-BN20 scores for motor dysfunction, fatigue, hair loss, leg weakness, and bladder control. These symptoms worsened 7 days after surgery compared to before surgery (*p* = 0.03, *p *= 0.048, *p* = 0.035, *p *= 0.001, and *p* = 0.014, respectively). One month after surgery, dyspnea and bladder control worsened (*p *= 0.007) and (*p* = 0.007), respectively. However, seizure improved one month after surgery (*p* = 0.009). Three months after surgery, skin pruritus worsened (*p* = 0.006). As shown in Table 2.

**Table 2:** Baseline QLQ-C30 and QLQ-BN20 scores and postoperative changes in patients with glioma

All subscales		Preop Mean (SD) & Medium (IQR) (*n *= 98)	7 days postop Mean (SD) & Medians (IQR) (*n* = 88)	u1	p2	One month postop Mean (SD) & Medium (IQR) (*n* = 45)	u2	p2	Three months postop Mean (SD) & Medium (IQR) (*n* = 33)	u3	p3
QLQ-C30	GHS	69.1 ± 22.7 66.7 (50, 83.3)	59.2 ± 25.7 58.3 (50, 75)	−2.541	0.011*	72.4 ± 21.2 70.8 (58.3, 91.7)	0.678	0.498	71.2 ± 20.9 66.7 (58.3, 83.3)	0.448	0.654
	PF	87.4 ± 20.7 100 (80, 100)	66.4 ± 29.5 73.3 (46.7, 93.3)	−5.753	< 0.001*	83.6 ± 24.7 93.3 (73.3, 100)	−0.955	0.340	84.9 ± 25.9 93.3 (83.3, 100)	−0.639	0.523
	RF	79.6 ± 28.6 100 (66.7, 100)	60.8 ± 31.1 66.7 (33.3, 95.8)	−4.445	< 0.001*	75.9 ± 30.1 83.3 (66.7, 100)	−0.905	0.365	83.3 ± 28.0 100 (83.3, 100)	0.752	0.452
	EF	84.4 ± 15.4 83.3 (75, 100)	86.0 ± 16.5 91.7 (75, 100)	0.987	0.323	86.1 ± 13.5 83.3 (83.3, 100)	0.528	0.598	83.6 ± 19.6 91.7 (75, 100)	0.095	0.924
	CF	79.4 ± 21.8 83.3 (66.7, 100)	70.3 ± 28.2 83.3 (50, 100)	−2.116	0.034*	76.3 ± 27.6 83.3 (66.7, 100)	−0.227	0.820	79.3 ± 27.6 83.3 (66.7, 100)	0.583	0.560
	SF	82.8 ± 21.3 100 (66.7, 100)	79.0 ± 25.2 83.3 (66.7, 100)	−0.889	0.374	78.2 ± 25.8 83.3 (66.7, 100)	−0.982	0.326	77.8 ± 26.2 83.3 (66.7, 100)	−0.902	0.367
	FA	22.1 ± 24.3 11.1 (0, 33.3)	34.3 ± 25.4 33.3 (11.1, 52.8)	3.712	< 0.001*	19.5 ± 20.8 11.1 (0, 33.3)	−0.353	0.724	22.6 ± 22.3 22.2 (0, 33.3)	0.427	0.670
	NV	6.0 ± 12.3 0 (0, 0)	10.2 ± 19.8 0 (0, 16.7)	1.068	0.285	6.3 ± 16.8 0 (0, 0)	−0.230	0.818	4.0 ± 9.3 0 (0, 0)	−0.684	0.494
	PA	12.9 ± 22.3 0 (0, 16.7)	20.3 ± 21.2 16.7 (0, 33.3)	3.359	0.001*	7.4 ± 14.5 0 (0, 16.7)	−1.219	0.223	10.1 ± 23.2 0 (0, 16.7)	−0.933	0.351
	DY	3.4 ± 11.2 0 (0, 0)	4.6 ± 11.5 0 (0, 0)	0.925	0.335	9.6 ± 16.9 0 (0, 33.3)	2.720	0.007*	5.1 ± 14.7 0 (0, 0)	0.512	0.609
	IN	11.2 ± 21.9 0 (0, 33.3)	13.6 ± 20.6 0 (0, 33.3)	1.275	0.202	9.6 ± 19.6 0 (0, 0)	−0.394	0.694	13.1 ± 23.5 0 (0, 33.3)	0.329	0.743
	AL	14.3 ± 25.3 0 (0, 33.3)	21.6 ± 29.5 0 (0, 33.3)	1.891	0.059	12.6 ± 25.9 0 (0, 16.7)	−0.658	0.511	10.1 ± 17.6 0 (0, 33.3)	−0.510	0.610
	CO	9.9 ± 21.0 0 (0, 0)	17.8 ± 25.7 0 (0, 33.3)	2.466	0.014*	16.3 ± 27.2 0 (0, 33.3)	1.462	0.144	17.2 ± 27.8 0 (0, 33.3)	1.593	0.111
	DI	4.4 ± 12.3 0 0(, 0)	0.8 ± 5.0 0 (0, 33.3)	−2.572	0.01*	2.2 ± 8.4 0 (0, 0)	−1.019	0.308	8.1 ± 14.5 0 (0, 16.7)	1.616	0.106
	FD	24.2 ± 27.4 33.3 0(, 33.3)	24.6 ± 29.7 0 (0, 33.3)	−0.108	0.914	31.9 ± 32.5 33.3 (0, 33.3)	1.261	0.207	35.4 ± 34.3 33.3 (0, 66.7)	1.614	0.107
QLQ-BN20	FU	18.3 ± 15.7 16.7 (8.3, 33.3)	18.5 ± 17.9 16.7 (0, 33.3)	−0.336	0.737	17.8 ± 19.7 16.7 (0, 25)	−0.725	0.468	15.4 ± 17.7 8.3 (0, 20.8)	−1.232	0.218
	VD	10.1 ± 15.6 0 (0, 22.2)	13.4 ± 19.7 0 (0, 22.2)	0.840	0.401	15.1 ± 24.1 0 (0, 22.2)	0.852	0.394	12.8 ± 18.0 0 (0, 22.2)	0.855	0.393
	MD	12.4 ± 18.5 0 (0, 22.2)	20.8 ± 25.5 11.1 (0, 33.3)	2.173	0.030*	13.1 ± 22.6 0 (0, 22.2)	−0.121	0.904	17.8 ± 26.5 11.1 (0, 33.3)	0.894	0.371
	CD	14.4 ± 24.0 0 (0, 22.2)	21.5 ± 24.8 11.1 (0, 33.3)	2.560	0.010*	22.2 ± 27.8 11.1 (0, 38.9)	1.840	0.066	17.2 ± 21.4 11.1 (0, 33.3)	1.128	0.259
	HA	22.3 ± 26.2 33.3 (0, 33.3)	26.5 ± 24.8 33.3 (0, 33.3)	1.398	0.162	14.8 ± 25.2 0 (0, 33.3)	−1.933	0.053	15.2 ± 25.1 0 (0, 33.3)	−1.616	0.106
	SE	11.3 ± 24.5 0 (0, 0)	6.1 ± 17.2 0 (0, 0)	−1.504	0.133	1.5 ± 6.9 0 (0, 0)	−2.632	0.009*	6.1 ± 15.5 0 (0, 0)	−0.902	0.367
	DR	21.0 ± 25.6 0 (0, 33.3)	28.4 ± 27.5 33.3 (0, 33.3)	1.978	0.048*	16.3 ± 24.2 0 (0, 33.3)	−1.084	0.278	16.2 ± 22.2 0 (0, 33.3)	−0.859	0.390
	HL	8.3 ± 16.7 0 (0, 0)	3.8 ± 11.8 0 (0, 0)	−2.108	0.035*	3.7 ± 10.6 0 (0, 0)	−1.551	0.121	13.1 ± 22.0 0 (0, 33.3)	1.303	0.193
	IS	3.1 ± 9.7 0 (0, 0)	3.0 ± 10.9 0 (0, 0)	−0.294	0.769	5.9 ± 14.7 0 (0, 0)	1.132	0.257	14.1 ± 26.4 0 (0, 33.3)	2.731	0.006*
	WL	12.0 ± 20.5 0 (0, 33.3)	26.5 ± 32.0 16.7 (0, 33.3)	3.255	0.001*	21.5 ± 29.4 0 (0, 33.3)	1.882	0.060	16.2 ± 23.7 0 (0, 33.3)	0.980	0.327
	BC	1.0 ± 5.8 0 (0, 0)	3.0 ± 10.9 0 (0, 0)	1.467	0.014*	6.7 ± 16.8 0 (0, 0)	2.720	0.007*	5.1 ± 18.9 0 (0, 0)	1.435	0.151

*P < 0.05; Abbreviations: GHS = Global Health Status; PF = Physical Function; RF = Role Function; EF = Emotional Function; CF = Cognitive Function; SF = Social Function; FA = Fatigue; NV = Nausea and Vomiting; PA = Pain; DY = Dyspnea; IN = Insomnia; AL = Appetite Loss; CO = Constipation; DI = Diarrhea; FD = Financial Difficulties; FU = Future Uncertainty; VD = Visual Disturbance; MD = Motor Dysfunction; CD = Communication Deficit; HA = Headache; SE = Seizures; DR = Drowsiness; HL = Hair Loss; IS = Itching Skin; WL = Weakness of the Legs; BC = Bladder Control; Postop = Postoperation; IQR = Interquartile range; SD = Standard Deviation; Note: Domain composite scores are presented as primary outcomes; analyses of individual items are exploratory supplementary results. All abbreviations are defined at the bottom of the table and in the main text

### Preoperative QLQ-C30 and QLQ-BN20 scores and postoperative changes in patients with brain metastases.

For brain metastases patients, composite scores of physical, role and social functions decreased significantly seven days after surgery (80.2 ± 24.0 vs. 57.0 ± 32.0, u = −3.669, p < 0.001; 68.3 ± 30.8 vs. 50.4 ± 31.7, u = −2.512, p = 0.012; 84.9 ± 19.1 vs. 73.3 ± 24.7, u = −2.258, p = 0.024). Multiple symptom domains also presented notable changes. As supplementary findings, fatigue and diarrhea showed significant alterations postoperatively (*p *= 0.044, *p* = 0.007), and leg weakness measured by QLQ-BN20 was statistically different (*p* = 0.03). At the one-month follow-up, future uncertainty and headache were alleviated with reduced scores (*p *= 0.031), (*p* = 0.005). Meanwhile, cognitive function scores increased, reflecting better cognitive performance (*p* = 0.018). Dyspnea was significantly exacerbated at the three-month follow-up (*p* = 0.031). As shown in Table 3.

**Table 3:** Baseline QLQ-C30 and QLQ-BN20 scores and postoperative changes in patients with BM

All subscales		preop Mean (SD) & Medium (IQR) (*n* = 42)	7 days postop Mean (SD) & Medians (IQR) (*n* = 40)	u1	p2	One month postop Mean (SD) & Medians (IQR) (*n* = 21)	u2	p2	Three months postop Mean (SD) & Medians (IQR) (*n* = 13)	u3	p3
EORTC QLQ-C30	GHS	67.5 ± 23.6 66.7 (50, 83.3)	63.7 ± 21.8 66.7 (50, 87.5)	−0.843	0.399	69.8 ± 22.0 66.7 (50, 87.5)	0.316	0.752	78.5 ± 13.0 79.2 (66.7, 83.3)	1.412	0.158
	PF	80.2 ± 24.0 86.7 (71.7, 100)	57 ± 32.0 63.3 (33.3, 85)	−3.669	< 0.001*	80.6 ± 24.4 93.3 (70, 96.7)	−0.156	0.876	86.2 ± 29.6 100 (93.3, 100)	1.489	0.137
	RF	68.3 ± 30.8 66.7 (33.3, 100)	50.4 ± 31.7 50 (33.3, 79.2)	−2.512	0.012*	65.9 ± 35.5 66.7 (33.3, 100)	−0.121	0.904	78.2 ± 30.0 83.3 (75, 100)	1.044	0.297
	EF	82.9 ± 19.1 87.5 (72.9, 100)	84.6 ± 19.6 91.7 (75, 100)	0.319	0.750	81.8 ± 20.2 91.7 (66.7, 100)	−0.445	0.657	82.7 ± 25.1 91.7 (79.2, 100)	0.289	0.772
	CF	71.8 ± 19.3 66.7 (50, 83.3)	68.8 ± 28.3 66.7 (50, 100)	−0.109	0.913	81.8 ± 25.2 83.3 (75, 100)	2.362	0.018*	82.1 ± 23.0 83.3 (83.3, 100)	1.894	0.058
	SF	84.9 ± 19.1 83.3 (66.7, 100)	73.3 ± 24.7 83.3 (54.2, 100)	−2.258	0.024*	73.8 ± 27.2 66.7 (66.7, 100)	−1.728	0.084	82.1 ± 30.8 100 (75, 100)	0.620	0.535
	FA	23.8 ± 26.6 11.1 (0, 44.4)	35.6 ± 28.6 33.3 (11.1, 63.9)	2.015	0.044*	16.9 ± 22.4 11.1 (0, 33.3)	−0.886	0.376	17.1 ± 30.0 0 (0, 22.2)	−1.234	0.217
	NV	9.1 ± 17.3 0 (0, 16.7)	11.7 ± 21.1 0 (0, 29.2)	0.182	0.856	5.6 ± 11.0 0 (0, 8.3)	−0.533	0.594	9.0 ± 27.7 0 (0, 0)	−0.869	0.385
	PA	25.4 ± 28.6 16.7 (0, 37.5)	20 ± 28.0 0 (0, 33.3)	−1.170	0.242	15.9 ± 23.3 0 (0, 33.3)	−1.368	0.171	14.1 ± 23.4 0 (0, 16.7)	−1.331	0.183
	DY	4.8 ± 13.9 0 (0, 0)	5.8 ± 12.8 0 (0, 0)	0.658	0.510	6.4 ± 17.1 0 (0, 0)	0.290	0.772	18.0 ± 29.2 0 (0, 33.3)	2.157	0.031*
	IN	18.3 ± 25.7 0 (0, 33.3)	15 ± 26.1 0 (0, 33.3)	−0.827	0.408	9.5 ± 18.7 0 (0, 16.7)	−1.352	0.176	15.4 ± 29.2 0 (0, 33.3)	−0.597	0.550
	AL	20.6 ± 32.1 0 (0, 33.3)	21.7 ± 32.5 0 (0, 33.3)	0.162	0.871	17.5 ± 22.7 0 (0, 33.3)	0.126	0.900	20.5 ± 32.0 0 (0, 33.3)	0.081	0.936
	CO	13.5 ± 26.6 0 (0, 33.3)	24.2 ± 35.4 0 (0, 33.3)	1.450	0.147	15.9 ± 20.1 0 (0, 33.3)	1.070	0.285	15.4 ± 37.6 0 (0, 0)	−0.546	0.585
	DI	7.1 ± 17.3 0 (0, 0)	0 ± 0 0 (0, 0)	−2.681	0.007*	6.4 ± 17.1 0 (0, 0)	−0.230	0.818	12.8 ± 21.7 0 (0, 33.3)	1.081	0.280
	FD	30. 2 ± 31.1 33.3 (0, 41.7)	27.5 ± 30.1 33.3 (0, 33.3)	−0.386	0.699	38.1 ± 30.3 33.3 (16.7, 66.7)	1.075	0.283	25.6 ± 20.0 33.3 (0, 33.3)	−0.159	0.873
EORTC QLQ-BN20	FU	23.6 ± 18.4 20.8 (8.3, 41.7)	16.9 ± 17.9 16.7 (2.1, 22.9)	−1.719	0.086	14.7 ± 19.3 8.3 (0, 25)	−2.155	0.031*	17.3 ± 16.5 8.3 (8.3, 25.0)	−1.016	0.310
	VD	16.7 ± 18.7 11.1 (0, 22.2)	19.2 ± 20.9 11.1 (0, 33.3)	0.457	0.647	19.1 ± 20.5 11.1 (0, 33.3)	0.394	0.693	17.1 ± 20.6 11.1 (0, 27.8)	0.010	−0.992
	MD	18.5 ± 23.1 11.1 (0, 33.3)	22.8 ± 25.8 11.1 (2.8, 22.2)	0.970	0.332	13.2 ± 19.1 11.1 (0, 22.2)	−0.783	0.434	17.1 ± 31.3 0 (0, 22.2)	−0.904	0.366
	CD	11.9 ± 21.3 0 (0, 13.9)	11.1 ± 18.1 0 (0, 19.4)	−0.116	0.907	9.0 ± 12.0 0 (0, 11.1)	0.203	0.839	12.8 ± 13.5 11.1 (0, 27.8)	1.056	0.291
	HA	31.0 ± 24.9 33.3 (0, 33.3)	25.0 ± 25.9 33.3 (0, 33.3)	−1.100	0.271	14.3 ± 24.9 0 (0, 33.3)	−2.823	0.005*	30.8 ± 28.7 33.3 (0, 33.3)	−0.196	0.884
	SE	4.8 ± 15.7 0 (0, 0)	3.3 ± 12.6 0 (0, 0)	−0.345	0.730	0 ± 0 0 (0, 0)	−1.449	0.147	5.1 ± 12.5 0 (0, 0)	0.513	0.608
	DR	19.1 ± 22.3 0 (0, 33.3)	25 ± 27.0 33.3 (0, 33.3)	0.917	0.359	11.1 ± 24.3 0 (0, 16.7)	−1.736	0.083	23.1 ± 37.0 0 (0, 33.3)	−0.244	0.807
	HL	6.4 ± 18.4 0 (0, 0)	3.3 ± 10.1 0 (0, 0)	−0.376	0.707	3.2 ± 10.0 0 (0, 0)	−0.361	0.718	7.7. ± 14.6 0 (0, 16.7)	0.841	0.400
	IS	8.7 ± 20.9 0 (0, 0)	4.2 ± 11.2 0 (0, 0)	−0.878	0.380	11.1 ± 21.9 0 (0, 16.7)	0.476	0.634	10.3 ± 16.0 0 (0, 33.3)	0.772	0.440
	WL	16.7 ± 23.6 0 (0, 33.3)	31.7 ± 32.9 33.3 (0, 58.3)	2.172	0.030*	15.9 ± 20.1 0 (0, 33.3)	0.109	0.913	18.0 ± 32.2 0 (0, 33.3)	−0.267	0.789
	BC	4.8 ± 13.9 0 (0, 0)	5.8 ± 16.7 0 (0, 0)	0.123	0.902	7.9 ± 18.0 0 (0, 0)	0.768	0.442	10.3 ± 28.5 0 (0, 0)	0.394	0.694

*P < 0.05; Abbreviations: GHS = Global Health Status; PF = Physical Function; RF = Role Function; EF = Emotional Function; CF = Cognitive Function; SF = Social Function; FA = Fatigue; NV = Nausea and Vomiting; PA = Pain; DY = Dyspnea; IN = Insomnia; AL = Appetite Loss; CO = Constipation; DI = Diarrhea; FD = Financial Difficulties; FU = Future Uncertainty; VD = Visual Disturbance; MD = Motor Dysfunction; CD = Communication Deficit; HA = Headache; SE = Seizures; DR = Drowsiness; HL = Hair Loss; IS = Itching Skin; WL = Weakness of the Legs; BC = Bladder Control; Postop = Postoperation; IQR = Interquartile range; SD = Standard Deviation;

### Factors Associated with HRQoL in patients with glioma during follow-up.

The following MLR results include both domain composite scores and individual item analyses, and the main interpretations are based on domain-level indicators. Multiple factors were found to be associated with quality of life, but not all of them can be presented. Therefore, we have selected the most valuable indicators in clinical treatment and nursing and analyzed those with significant differences based on clinical experience. The results of the MLR did not indicate multicollinearity between the included variables. Table 4 shows the estimates for the association between the selected variables and fatigue, motor dysfunction, communication deficit, and leg weakness 7 days after surgery. For example, age (B = 0.6, 95% CI 0.1 to 1.1, P < 0.05), years of education (B = −1.4, 95% CI −2.7 to −0.1, P < 0.05), and right-sided tumor (B = −20.0, 95% CI −30.5 to −9.6, P < 0.05) were independently associated with communication deficit. The standardized coefficients further indicated that these factors exerted moderate effect sizes on communication deficit. All included variables showed no multicollinearity (VIF < 5). Supplementary Table 1 shows the estimates of the association between the selected variables and physical function, nausea and vomiting, loss of appetite, future uncertainty, motor dysfunction, communication deficit, and leg weakness one month after surgery. Multiple lobes (−27.3; 95% CI −46.6 to −8.0), subtotal resection (−29.4; 95% CI −57.9 to −0.9), and GTR (−23.5; 95% CI −41.9 to −5.2) were significantly associated with worse motor dysfunction at a statistically and clinically relevant level. The occipital lobe (44.9; 95% CI 4.6 to 85.3) was significantly associated with better motor dysfunction. Supplementary Table 2 shows the estimates of the association between the selected variables and physical function, role function, emotional function, cognitive function, social function, financial difficulties, and leg weakness three months after surgery. Female gender (23.1; 95% CI 1.1 to 45.0), right hemisphere (34.3; 95% CI 12.9 to 55.8), and subtotal resection (72.3; 95% CI 20.7 to 123.8) were significantly associated with better cognitive function at a statistically and clinically relevant level, whereas grade 4 glioma (−64.2; 95% CI −120.4 to −8.1) was significantly associated with worse cognitive function at a statistically and clinically relevant level.

**Table 4:** The MLR results of selected HRQoL scales for patients with glioma (7 days after surgery)

Variable	GHS B (SE), VIF, P β (95% CI)	FA B (SE), VIF, P β (95% CI)	MD B (SE), VIF, P β (95% CI)	CD B (SE), VIF, P β (95% CI)	WL B (SE), VIF, P β (95% CI)
Male	Ref	Ref	Ref	Ref	Ref
Female	−2.5 (6.2), 1.29, 0.69 −0.05, (−14.8, 9.9)	3.8 (5.7), 1.29, 0.5 0.07 (−7.5, 15.2)	11.2 (5.1), 1.29, 0.03 0.21 (1.0, 21.4)*	−3.6 (5.06), 1.29, 0.48 −0.07 (−13.7, 6.5)	14.2 (6.6), 1.29, 0.03 0.22 (1.1, 27.3)*
Age, year	−0.2 (0.3), 2.1, 0.45 −0.12 (−0.8, 0.4)	0.1 (0.28), 2.07, 0.69 0.06 (−0.4, 0.7)	0.4 (0.25), 2.07, 0.08 0.22 (−0.1, 0.9)	0.6 (0.25), 2.1, 0.02 0.3 (0.1, 1.1)*	0.7 (0.32), 2.1, 0.04 0.27 (0.03, 1.3)*
BMI, kg/m2	1.0 (0.85), 1.15, 0.26 0.13 (−0.7, 2.6)	0.5 (0.78), 1.15, 0.51 0.07 (−1.0, 2.1)	−1.2 (0.7), 1.15, 0.104 −0.15 (−2.5, 0.2)	−1.2 (0.69), 1.15, 0.09 −0.16 (−2.6, 0.2)	−0.8 (0.9), 1.15, 0.37 −0.09 (−2.6, 1.0)
Education, year	−0.7 (0.8), 1.15, 0.36 −0.11 (−2.3, 0.9)	−0.9 (0.73), 1.15, 0.23 −0.13 (−2.3, 0.6)	0.1 (0.66), 1.15, 0.86 0.02 (−1.2, 1.4)	−1.4 (0.65), 1.15, 0.036 −0.2 (−2.7, −0.1)*	0.5 (0.85), 1.15, 0.53 0.06 (−1.2, 2.2)
Biopsy only	Ref	Ref	Ref	Ref	Ref
Sub-resection	6.7 (19.5), 2.4, 0.73 0.06 (−32.2, 45.6)	−18.9 (17.9), 2.4, 0.295 −0.16 (−54.8, 16.9)	6.8 (16.1), 2.4, 0.68 0.06 (−25.3, 38.8)	−4.4 (15.9), 2.4, 0.79 −0.04 (−36.2, 27.5)	−41.0 (20.6), 2.4, 0.05 −0.28 (−82.2, 0.3)
Total-resection	0.91 (7.9), 1.54, 0.9 0.02 (−14.9, 16.7)	1.2 (7.3), 1.54, 0.87 0.02 (−13.4, 15.8)	−5.6 (6.5), 1.54, 0.4 −0.09 (−18.6, 7.5)	4.4 (6.5), 1.54, 0.5 0.07 (−8.6, 17.3)	−11.5 (8.4), 1.54, 0.18 −0.15 (−28.3, 5.3)
Grade					
I	Ref	Ref	Ref	Ref	Ref
II	−31.2 (26.5), 21.8, 0.24 −0.59 (−84.3, 21.8)	1.4 (24.4), 21.8, 0.95 0.03 (−47.4, 50.3)	3.3 (21.9), 21.8, 0.88 0.06 (−40.4, 47.0)	11.5 (21.7), 21.8, 0.6 0.22 (−31.9, 54.9)	9.5 (28.1), 21.8, 0.74 0.14 (−46.7, 65.7)
III	−32.0 (27.0), 20.3, 0.24 −0.57 (−86.1, 22.0)	5.8 (24.9), 20.3, 0.82 0.1 (−44.0, 55.5)	8.2 (22.3), 20.3, 0.72 0.14 (−36.4, 52.7)	21.9 (22.1), 20.3, 0.326 0.39 (−22.3, 66.1)	28.1 (28.6), 20.3, 0.33 0.39 (−29.2, 85.3)
IV	−28.5 (26.4), 22.3, 0.29 −0.55 (−81.3, 24.3)	1.3 (24.3), 22.3, 0.96 0.03 (−47.3, 49.9)	2.5 (21.8), 22.3, 0.91 0.05 (−41.0, 46.0)	3.8 (21.6), 22.3, 0.86 0.07 (−39.4, 47.0)	13.6 (28.0), 22.3, 0.63 0.2 (−42.3, 69.5)
Side of the tumor					
Left hemisphere	Ref	Ref	Ref	Ref	Ref
Right hemisphere	−2.2 (6.4), 1.4, 0.73 −0.04 (−15.0, 10.6)	−2.0 (5.9), 1.4, 0.73 −0.04 (−13.8, 9.7)	−2.8 (5.3), 1.4, 0.6 −0.05 (−13.3, 7.8)	−20.0 (5.23), 1.4, 0 −0.4 (−30.5, −9.6)*	0.1 (6.8), 1.4, 0.99 0.001 (−13.5, 13.6)
Bilateral	7.5 (26.8), 2.4, 0.78 0.05 (−46.0, 61.1)	37.1 (24.7), 2.35, 0.14 0.22 (−12.2, 86.4)	−42.9 (22.1), 2.35, 0.057 −0.26 (−87.1, 1.2)	−13.2 (21.9), 2.35, 0.55 −0.08 (−57.0, 30.7)	51.5 (28.4), 2.35, 0.08 0.25 (−5.3, 108.3)
Tumor Location					
Frontal lobe	Ref	Ref	Ref	Ref	Ref
Temporal lobe	3.1 (7.9), 1.35, 0.69 0.05 (−12.6, 18.9)	−3.7 (7.3), 1.35, 0.61 −0.06 (−18.3, 10.8)	−13.0 (6.5), 1.35, 0.05 −0.2 (−26.0, 0.1)	7.1 (6.5), 1.35, 0.27 0.11 (−5.8, 20.0)	−11.0 (8.37), 1.35, 0.19 −0.14 (−27.8, 5.7)
Parietal lobe	14.0 (9.3), 1.16, 0.14 0.17 (−4.6, 32.5)	−17.4 (8.55), 1.16, 0.04 −0.21 (−34.5, −0.3)*	−2.1 (7.65), 1.16, 0.78 −0.03 (−17.4, 13.2)	−0.2 (7.59), 1.16, 0.98 −0.003 (−15.4, 14.9)	−12.9 (9.8), 1.16, 0.2 −0.13 (−32.5, 6.8)
Occipital lobe	−8.4 (15.6), 1.18, 0.59 −0.06 (−39.5, 22.7)	−16.7 (14.3), 1.18, 0.25 −0.12 (−45.3, 12.0)	−7.6 (12.8), 1.18, 0.56 −0.06 (−33.2, 18.1)	3.2 (12.7), 1.18, 0.81 0.02 (−22.3, 28.6)	−18.4 (16.5), 1.18, 0.27 −0.1 (−51.3, 14.6)
Insula lobes	-	-	-	-	-
Multiple Lobes	0.2 (8.6), 1.28, 0.98 0.003 (−17.0, 17.4)	−0.1 (7.9), 1.28, 0.99 −0.001 (−16.0, 15.8)	−10.5 (7.11), 1.28, 0.15 −0.14 (−24.7, 3.7)	4.8 (7.1), 1.28, 0.5 0.07 (−9.3, 18.9)	−17.2 (9.1), 1.28, 0.06 −0.19 (−35.5, 1.1)
Others	21.8 (18.8), 1.16, 0.25 0.13(−15.8, 59.3)	−34.8 (17.3), 1.16, 0.049 −0.21 (−69.4, −0.2)*	−18.0 (15.5), 1.16, 0.25 −0.11 (−49.0, 12.9)	2.9 (15.4), 1.16, 0.85 0.02 (−27.9, 33.6)	−35.3 (19.9), 1.16, 0.08 −0.17 (−75.1, 4.5)
Newly diagnosis	Ref	Ref	Ref	Ref	Ref
Recurrence	3.5 (7.6), 1.3, 0.65 0.06 (−11.7, 18.8)	3.6 (7.03), 1.26, 0.61 0.06 (−10.4, 17.7)	−1.4 (6.29), 1.26, 0.83 −0.02 (−14.0, 11.2)	4.6 (6.25), 1.26, 0.47 0.07 (−7.9, 17.1)	−3.0 (8.1), 1.26, 0.7 −0.04 (−19.2, 13.2)
KPS	0.4 (0.13), 1.23, 0.01 0.38 (0.2, 0.7)	−0.5 (0.12), 1.23, 0 −0.43 (−0.7, −0.2)*	−0.6 (0.11), 1.23, 0 −0.53 (−0.8, −0.4)*	−0.02 (0.11), 1.23, 0.85 −0.02 (−0.2, 0.2)	−0.4 (0.14), 1.23, 0.01 −0.3 (−0.7, −0.2)*

*P < 0.05; Abbreviations: Ref, reference group; B = Unstandardized regression coefficient; SE = Standard error; β = Standardized regression coefficient; 95% CI = 95% confidence interval; VIF = Variance Inflation Factor. GHS = Global Health Status; FA = Fatigue; MD = Motor Dysfunction; CD = Communication Deficit; WL = Weakness of the Legs;

### Factors Associated with HRQoL in Patients with BM During Follow-up.

The results of the MLR did not indicate multicollinearity between the included variables. Table 5 shows the estimates for the association between the selected variables and future uncertainty and headache 7 days after surgery. For example, age (B = −0.9, 95% CI −1.6 to −0.3) and parietal lobe tumor (B = −26.1, 95% CI −48.7 to −3.4) were independently associated with postoperative headache (both P < 0.05). Combined unstandardized and standardized regression coefficients, we confirmed the clinical meaningful effect sizes of the above associations, and no multicollinearity was detected in the regression model (VIF < 5). The same table presents the relationships between variables and constipation as well as visual disturbance at the one-month follow-up. Age (−1.4; 95% CI −2.6 to −0.2), education (−3.0; 95% CI −5.2 to −0.8) and GTR (−61.1; 95% CI −95.2 to −26.9) correlated with worse constipation. In contrast, BMI (5.3; 95% CI 3.4 to 7.2) and parietal lobe were linked to better constipation.

**Table 5:** The MLR results of selected HRQoL scales for patients with brain metastases

Variable	7 days postop		One month postop		Three months postop			
	FU B (SE), VIF, P β (95% CI)	HA B (SE), VIF, P β (95% CI)	CO B (SE), VIF, P β (95% CI)	VD B (SE), VIF, P β (95% CI)	FA B (SE), VIF, P β (95% CI)	NV B (SE), VIF, P β (95% CI)	VD B (SE), VIF, P β (95% CI)	HL B (SE), VIF, P β (95% CI)
Male	Ref	Ref	Ref	Ref	Ref	Ref	Ref	Ref
Female	6.1 (5.6), 1.35, 0.3 0.2 (−5.4, 17.6)	−8.8 (8.01), 1.35, 0.28 −0.17 (−25.3, 7.7)	−10.1 (7.36), 2.24, 0.2 −0.23 (−26.8, 6.5)	−15.3 (10), 2.24, 0.16 −0.34 (−37.9, 7.3)	−117.7 (52.9), 75.0 −1.89(−286.2, 50.7)	54.4 (33.5), 75.0, 0.2 0.94 (−52.1, 160.8)	−56.1 (17.1), 75.0, 0.04 −1.3 (−110.4, −1.8)*	32.2 (13.6), 75.0, 0.1 1.1 (−11.1, 75.6)
Age, year	−1.0 (0.22), 1.38, 0 −0.69 (−1.5, −0.5)*	−0.9 (0.32), 1.38, 0.01 −0.44 (−1.6, −0.3)*	−1.4 (0.53), 3.64, 0.03 −0.56 (−2.6, −0.2)*	−0.6 (0.72), 3.64 −0.24 (−2.2, 1.0)	1.1 (1.03), 40.4, 0.35 0.69 (−2.1, 4.4)	−1.0 (0.65), 40.4, 0.22 −0.65 (−3.1, 1.1)	−0.2 (0.33), 40.4, 0.53 −0.21 (−1.3, 0.8)	−0.2 (0.27), 40.4, 0.42 −0.31 (−1.1, 0.6)
BMI, kg/m2	−0.4 (0.82), 1.52, 0.59 −0.09 (−2.1, 1.2)	−1.2 (1.18), 1.52, 0.34 −0.16 (−3.6, 1.3)	5.3 (0.81), 1.84, 0 0.98 (3.4, 7.2)*	−1.0 (1.14), 1.84 −0.17 (−3.5, 1.6)	−0.1 (1.48), 3.6, 0.96 −0.01 (−4.8, 4.6)	0.5 (0.94), 3.57, 0.66 0.06 (−2.5, 3.4)	−0.2 (0.48), 3.57, 0.67 −0.04 (−1.7, 1.3)	−0.3 (0.38), 3.57, 0.45 −0.09 (−1.5, 0.9)
Education, year	−1.2 (0.93), 1.8, 0.22 −0.22 (−3.1, 0.7)	0.9 (1.34), 1.8, 0.49 0.12 (−1.8, 3.7)	−3.0 (0.97), 2.0, 0.01 −0.5 (−5.2, −0.8)*	−0.9 (1.32), 2.0, 0.51 −0.15 (−3.9, 2.1)	0.04 (2.3), 6.1, 0.99 0.004 (−7.4, 7.5)	−1.2 (1.47), 6.1, 0.47 −0.14 (−5.9, 3.5)	−4.3 (0.75), 6.11, 0.01 −0.66 (−6.7, −1.9)*	2.9 (0.6), 6.11, 0.017 0.62 (1.0, 4.8)*
Biopsy only	Ref	Ref	Ref	Ref	Ref	Ref	Ref	Ref
Sub-resection	−10.0 (21.1), 2.0, 0.64 −0.09 (−53.4, 33.4)	24.8 (30.3), 2.0, 0.42 0.15 (−37.6, 87.2)	-	-	-	-	-	-
Total-resection	11.0 (11.3), 1.63, 0.34 0.16 (−12.2, 34.2)	−5.4 (16.2), 1.63, 0.74 −0.06 (−38.7, 27.9)	−61.1 (15.1), 2.1, 0.01 −0.7 (−95.2, −26.9)*	−40.4 (21.3), 2.27, 0.06 0.48 (−86.7, 6.0)	-	-	-	-
Side of the tumor								
Left hemisphere	Ref	Ref	Ref	Ref	Ref	Ref	Ref	Ref
Right hemisphere	−10.0 (6.22), 1.5, 0.12 −0.26 (−22.8, 2.8)	6.8 (8.9), 1.5, 0.45 0.12 (−11.6, 25.2)	−1.1 (6.2), 1.89, 0.85 −0.03 (−15.1, 12.8)	15.2 (8.4), 1.89, 0.1 0.38 (−3.7, 34.1)	−34.6 (18.9), 10.7, 0.2 −0.6 (−94.8, 25.7)	16.7 (12.0), 10.7, 0.26 0.3 (−21.4, 54.7)	−4.8 (6.1), 10.7, 0.49 −0.12 (−24.2, 146)	−0.5 (4.88), 10.7, 0.92 −0.02 (−16.1, 15.0)
Bilateral	−25.0 (20.8), 1.95, 0.24 −0.22 (−67.8, 17.9)	−27.8 (8.9), 1.9, 0.36 −0.17 (−89.4, 33.7)	-	-	-	-	-	-
Tumor Location								
Frontal lobe	Ref	Ref	Ref	Ref	Ref	Ref	Ref	Ref
Temporal lobe	−11.5 (11.3), 1.12, 0.32 −0.14 (−34.7, 11.7)	−15.2 (16.2), 1.12, 0.36 −0.13 (−48.5, 18.1)	−13.7 (12.3), 1.4, 0.3 −0.15 (−41.6, 14.2)	−6.0 (16.8), 1.4, 0.73 −0.06 (−43.9, 31.9)	−155.3 (69.4), 42.9, 0.11 −1.44 (−376.1, 65.4)	67.7 (43.8), 42.9, 0.2 0.68 (−71.8, 202.2)	−62.6 (22.3), 42.9, 0.07 −0.84 (−133.7, 8.5)	63.3 (17.9), 42.9, 0.04 1.2 (6.4, 120.1)*
Parietal lobe	−7.8 (7.66), 1.19, 0.32 −0.15 (−23.6, 8.0)	−26.1 (11.0), 1.19, 0.03 −0.34 (−48.7, −3.4)*	45.4 (9.27), 1.5, 0.01 0.68 (24.4, 66.4)*	23.8 (12.6), 1.5, 0.09 0.35 (−4.7, 52.3)	-	-	-	-
Occipital lobe	−7.3 (7.3), 1.25, 0.33 −0.15 (−22.2, 7.7)	16.1 (10.4), 1.25, 0.14 0.23 (−5.4, 37.6)	9.7 (8.2), 1.67, 0.28 0.17 (−8.8, 28.2)	29.7 (11.1), 1.67, 0.03 0.52(4.5, 54.9)*	−13.7 (11.4), 2.14, 0.32 −0.17 (−50.1, 22.6)	2.5 (7.22), 2.14, 0.76 0.03 (−20.5, 25.4)	24.9 (3.68), 2.14, 0.01 0.45 (13.1, 36.6)*	6.6 (2.94), 2.14, 0.11 0.17 (−2.7, 16.0)
Insula lobes	−9.9 (16.6), 1.24, 0.56 −0.09 (−44.1, 24.2)	−22.4 (23.8), 1.24, 0.36 −0.14 (−71.5, 26.7)	-	-	-	-	-	-
Multiple Lobes	−0.3 (8.5), 1.72, 0.97 −0.006 (−17.9, 17.3)	−3.7 (12.3), 1.7, 0.76 −0.05 (−29.0, 21.6)	−6.1 (7.47), 2.1, 0.43 −0.13 (−23.0, 10.8)	18.0 (10.1), 2.1, 0.11 0.38 (−4.9, 41.0)	−133.3 (65.2), 69.5, 0.13 −1.7 (−340.8, 74.2)	64.1 (41.2), 69.5, 0.2 0.87 (−67.0, 195.3)	−46.8 (21.0), 69.5, 0.11 −0.85 (−113.7, 20.0)	34.1 (16.8), 69.5, 0.14 0.88 (−19.3, 87.6)
Others	−2.0 (11.6), 1.72, 0.87 −0.03 (−25.8, 21.9)	32.3 (16.6), 1.7, 0.06 0.33 (−2.0, 66.5)	−17.3 (15.7), 2.2, 0.3 −0.2 (−52.9, 18.2)	45.3 (21.3), 2.3, 0.06 0.48 (−2.9, 93.6)	82.2 (21.0), 3.95, 0.03 0.76 (15.3, 149.2)*	100.1 (13.3), 4.0, 0.01 1.0 (57.7, 142.4)*	84.0 (6.78), 3.95, 0.001 1.13 (62.4, 105.5)*	12.1 (5.42), 5.95, 0.11 0.23 (−5.2, 29.3)

*P < 0.05; Abbreviations: Ref = Reference group; B = Unstandardized regression coefficient; SE = Standard error; β = Standardized regression coefficient; 95% CI = 95% confidence interval; VIF = Variance Inflation Factor. FU = Future Uncertainty; HA = Headache; CD = Communication Deficit; FA = Fatigue; NV = Nausea and Vomiting; VD = Visual Disturbance; HL = Hair Loss;

We also analyzed correlations between selected variables and fatigue, nausea and vomiting, visual disturbances, and hair loss at three months after surgery. Female gender (−56.1; 95% CI −110.4 to −1.8), education (−4.3; 95% CI −6.7 to −1.9), and GTR (−61.1; 95% CI −95.2 to −26.9) were significantly associated with worse visual disturbance. In contrast, occipital lobe (24.9; 95% CI 13.1 to 36.6) and other tumor locations were linked to better visual disturbance. Significant changes in fatigue and diarrhea were observed from baseline to 7 days after surgery in BM, while no significant associations were found in the MLR analysis.

### Other results

**Fig. 2:**
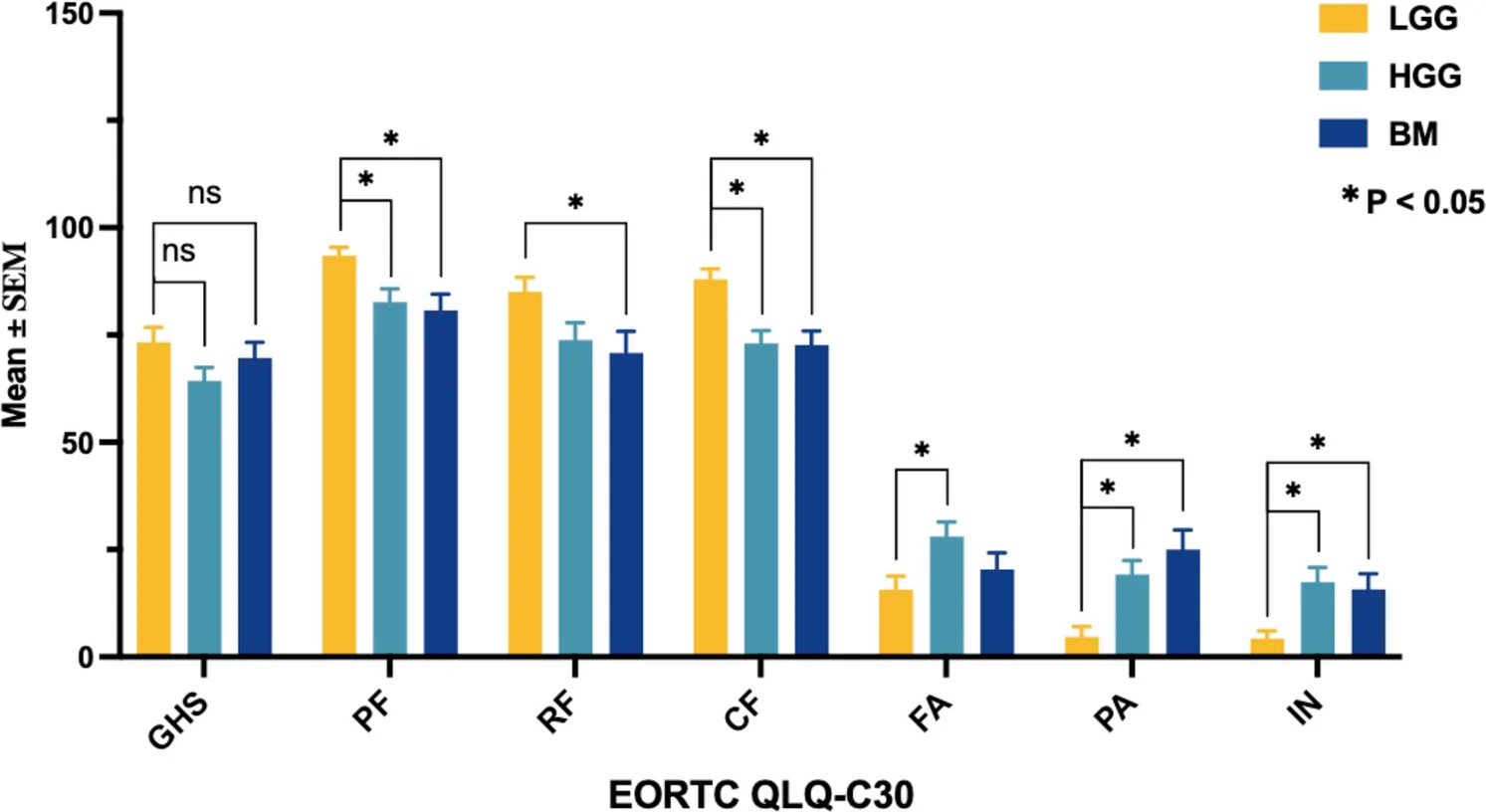
Baseline QLQ-C30 scores across LGG, HGG and BM. Abbreviations: LGG = low-grade glioma; HGG = high-grade glioma; BM = brain metastases; ns = no significant difference

**Fig. 3:**
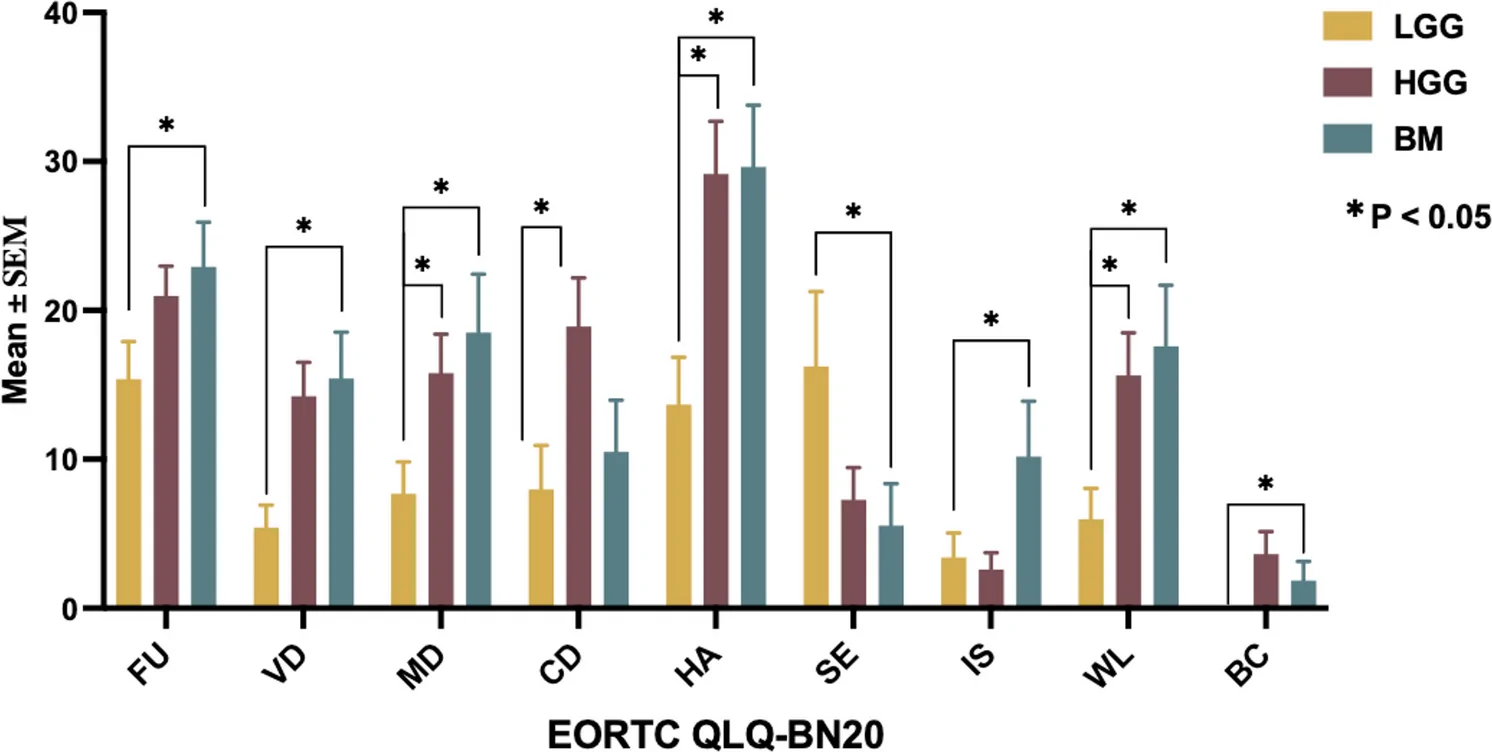
Baseline QLQ-BN20 scores across LGG, HGG and BM. Abbreviations: LGG = low-grade glioma; HGG = high-grade glioma; BM = Brain metastases; ns = no significant difference

**Fig. 4:**
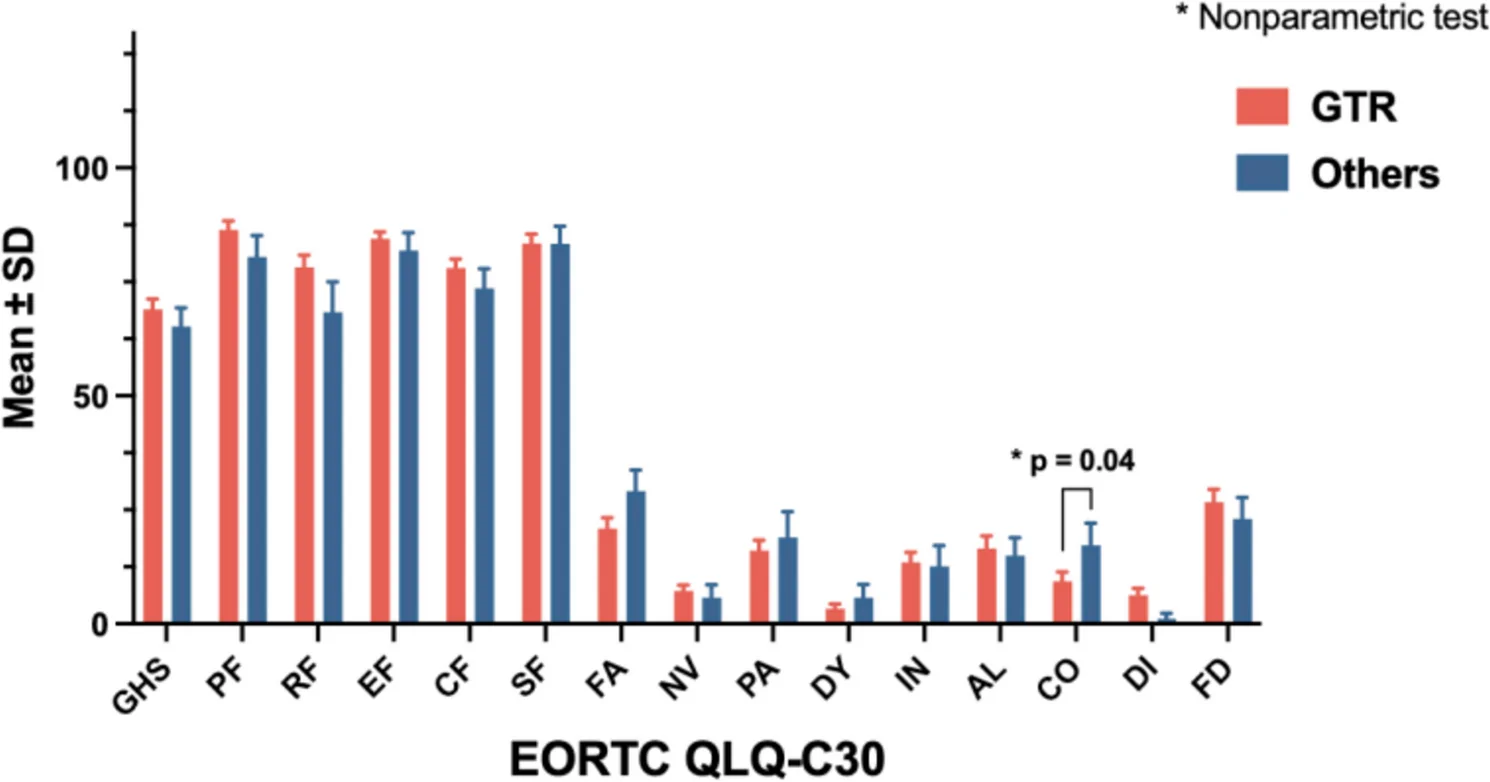
Baseline QLQ-C30 scores: GTR vs. biopsy or partial resection. Abbreviations: GTR = Gross total resection;

**Fig. 5:**
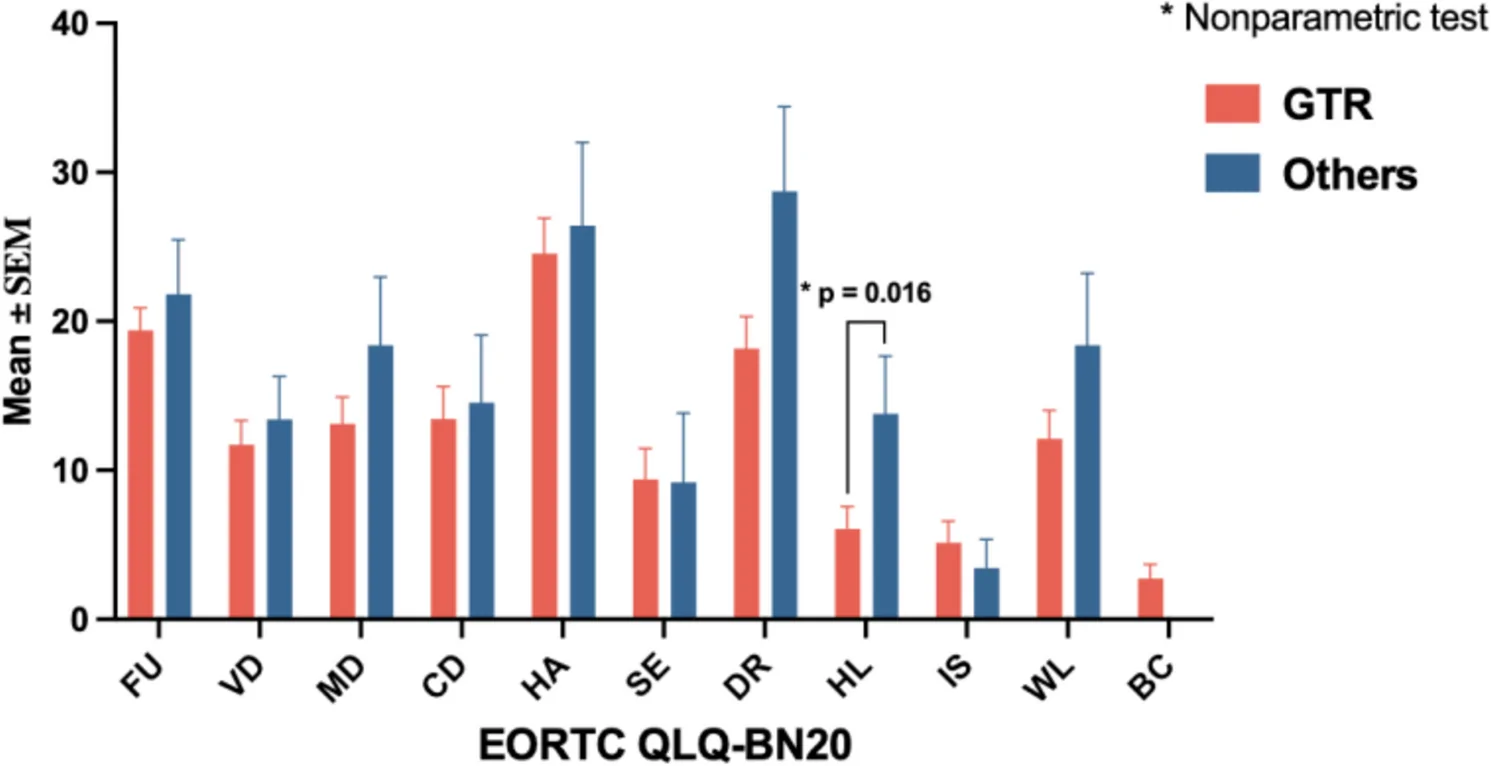
Baseline QLQ-BN20 scores: GTR vs. biopsy or partial resection. Abbreviations: GTR = Gross total resection;

**Fig. 6:**
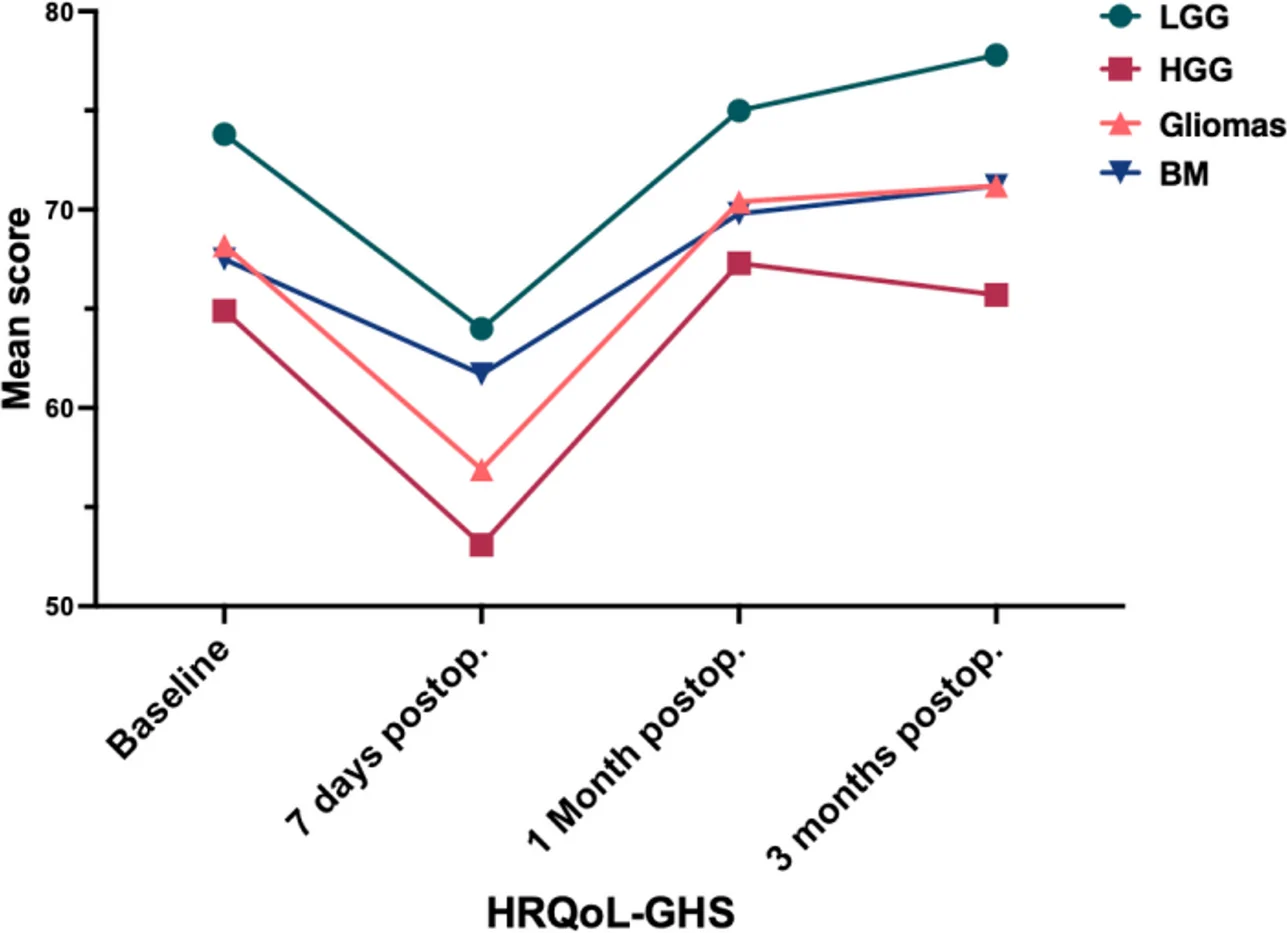
Changes in GHS during 3-month follow-up. Abbreviations: HRQoL-GHS = Health-related quality of life Global Health Status; LGG = low-grade glioma; HGG = high-grade glioma; BM = Brain metastases; Postop = Postoperation;

Baseline HRQoL scores differed significantly across low-grade glioma, high-grade glioma and brain metastases in physical function, cognitive function, pain, insomnia, motor disfunction, headache and weakness of the legs (Figs. 2–3). Significant disparities in constipation and hair loss were also observed between patients receiving GTR and those undergoing biopsy or partial resection (Figs. 4–5). Given the clinical heterogeneity between gliomas and BM, we further stratified analyses by tumor type, surgical approach and tumor laterality to compare baseline and postoperative HRQoL (Fig. 6).

**Fig. 7:**
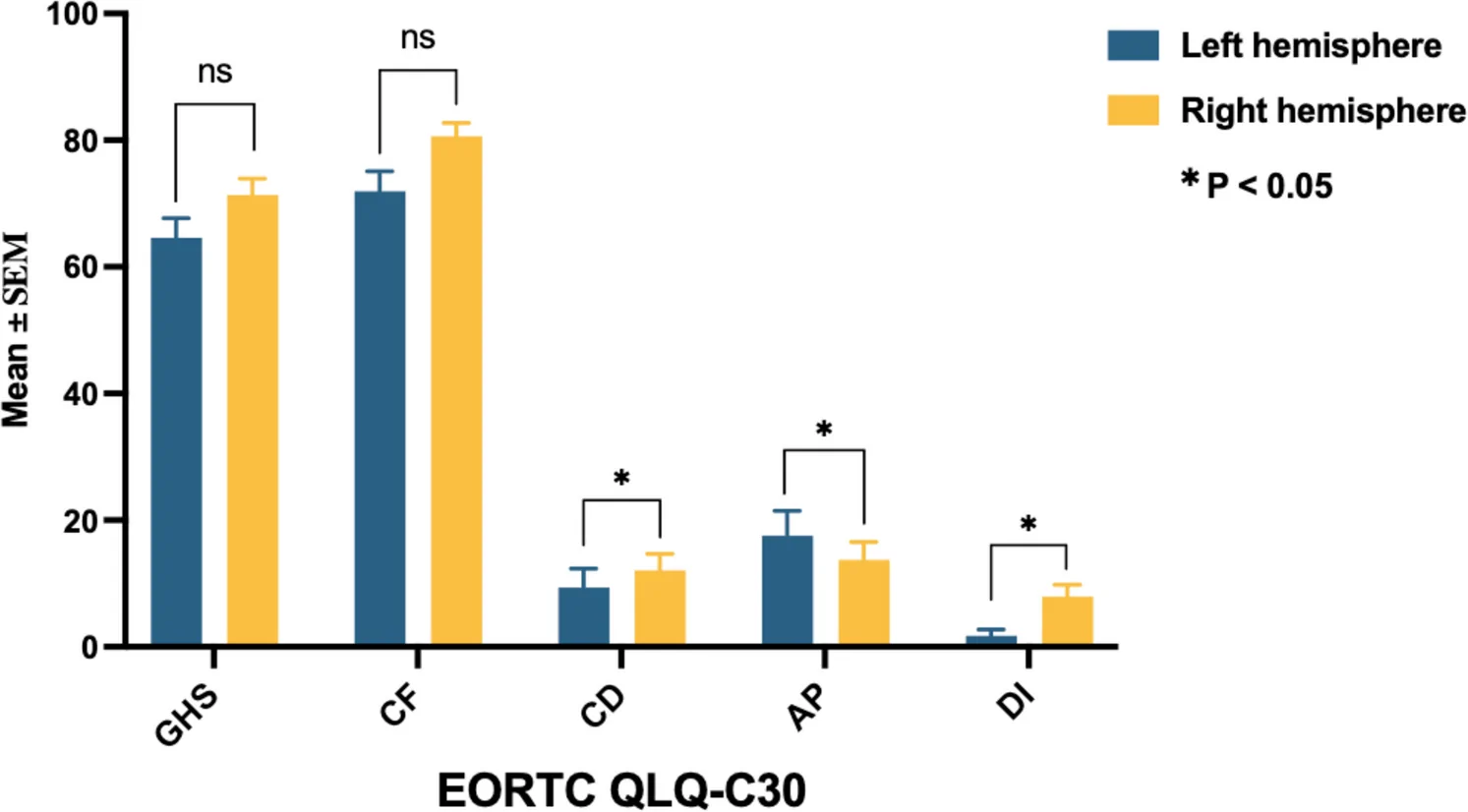
Baseline HRQoL scores: left vs. right hemisphere tumors. Abbreviations: ns = no significant difference

**Fig. 8:**
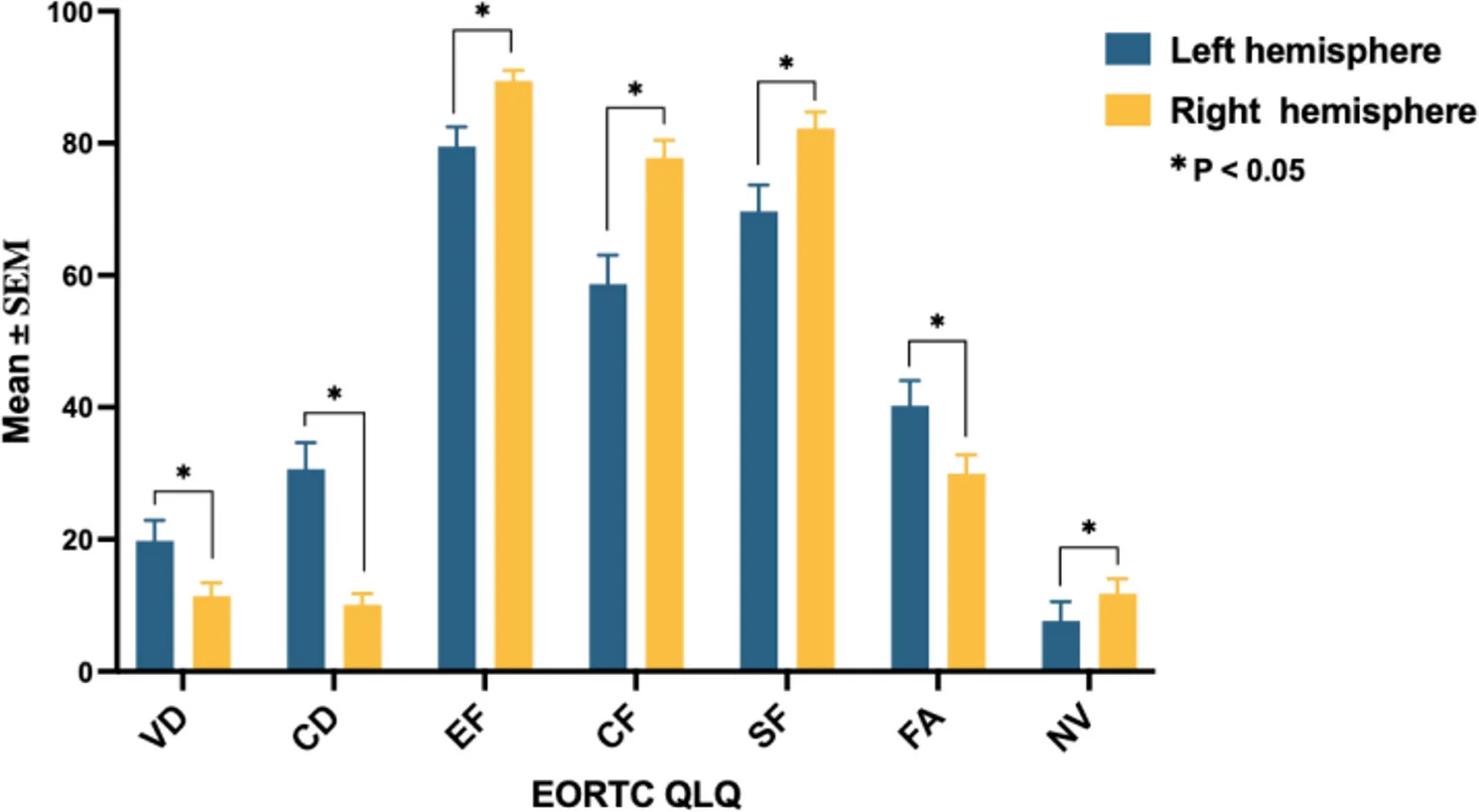
HRQoL scores at postoperative day 7: left vs. right hemisphere tumors

**Fig. 9:**
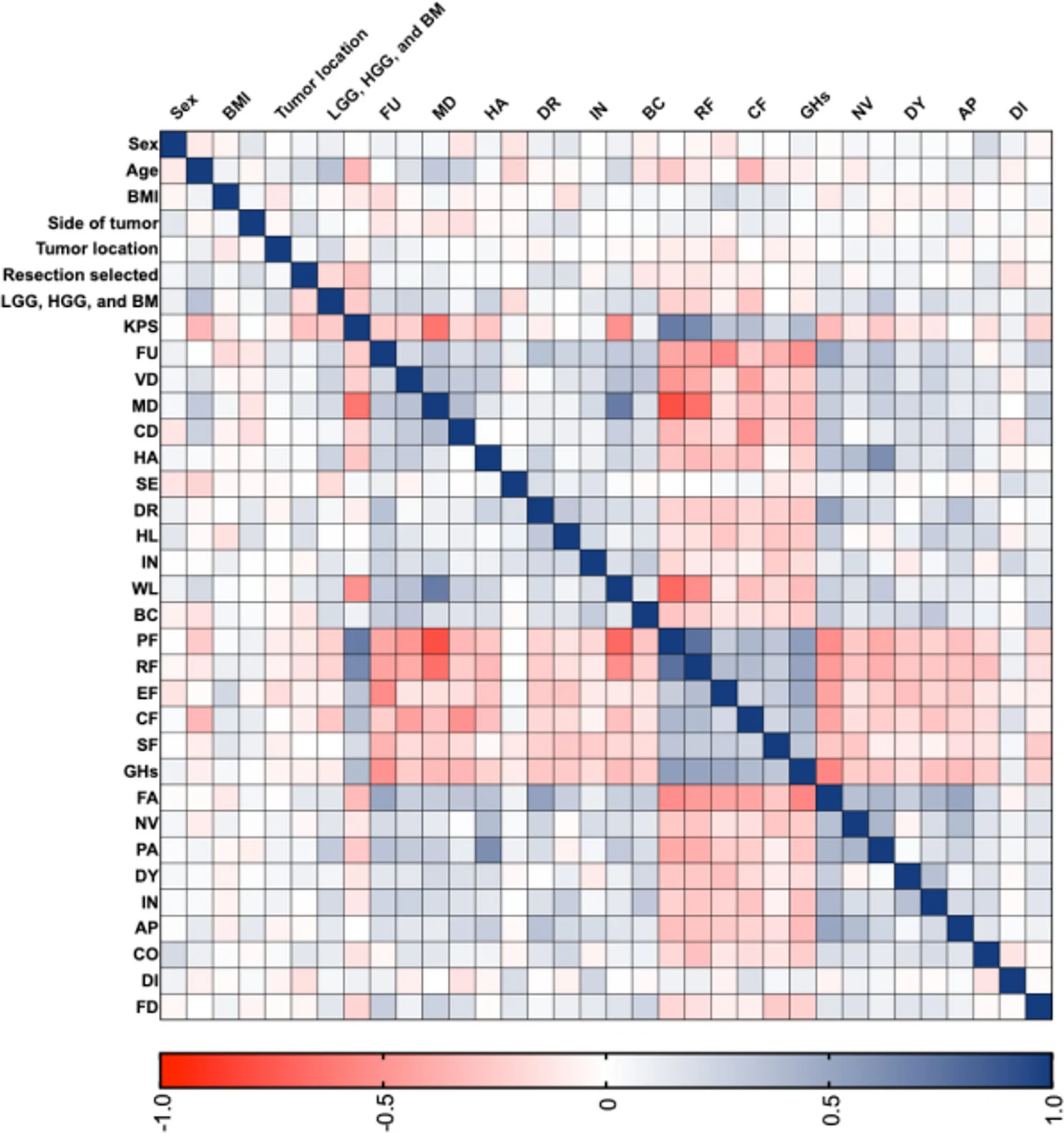
Correlations between clinical factors and HRQoL domains

Baseline scores of communication deficit, appetite loss and diarrhea varied significantly between left- and right-sided tumors (Fig. 7). At postoperative day 7, significant inter-group differences were detected in visual disturbance, communication deficit, emotional function, cognitive function, social function, fatigue, nausea and vomiting (Fig. 8). Global health status followed a V-shaped trend from baseline to one month postoperatively across all subgroups, with mild fluctuations persisting through the three-month follow-up (Fig. 6). KPS was negatively correlated with headache, fatigue and pain. Additional correlation results are presented in Fig. 9.

## Discussion

This prospective longitudinal study explored perioperative changes in health-related quality of life among patients with malignant brain tumors, including gliomas and brain metastases, over a 3-month postoperative period using the EORTC QLQ-C30 and QLQ-BN20 questionnaires [16, 17]. Overall, patients experienced obvious fluctuations in multiple HRQoL domains in the early postoperative phase. Given the distinct disease features and treatment backgrounds between gliomas and BM, we conducted combined and subgroup analyses to characterize their unique patient-reported outcome trajectories. The one-week postoperative time point was selected to capture acute functional changes and symptoms related to surgical trauma, which is a key component of perioperative assessment. Across all subgroups including low-grade glioma, high-grade glioma and BM, global health status presented a typical V-shaped trend: it declined sharply at postoperative day 7 and gradually recovered one month after surgery, with HGG patients showing a slower recovery pace [15].

For glioma patients, physical function, role function and multiple symptoms deteriorated significantly in the acute postoperative stage, before partial recovery at the one-month follow-up. Seizure-related symptoms were notably relieved after surgery, which is consistent with the clinical effect of tumor resection on epileptic foci. Postoperative seizure control is another notable advantage of tumor resection, and standardized anti-epileptic medication can further optimize this outcome [18]. For patients with brain metastases, physical, role and social function declined markedly shortly after surgery; meanwhile, psychological distress reflected by future uncertainty and headache gradually eased over time, accompanied by improved cognitive function. These subgroup differences reflect the inherent distinctions in disease nature, primary tumor background and overall physical condition between the two patient populations [5, 6].

Age showed distinct correlations with HRQoL indicators in glioma and brain metastases subgroups. Among glioma patients, advanced age was linked to milder communication deficits and lower extremity weakness after surgery. Combined with our baseline data and surgical inclusion criteria, this counterintuitive finding is largely attributed to selection bias. Elderly patients with severe preoperative neurological deficits or multiple comorbidities were generally not candidates for craniotomy. Thus, the elderly cohort enrolled in this study represented a relatively healthy group with better baseline neurological reserve [19, 20]. In contrast, older patients with brain metastases tended to report higher levels of future uncertainty and headache, which may be related to the psychological burden and complex disease course of metastatic tumor patients. These findings remind clinicians to implement age-stratified assessment and targeted supportive care for different MBT populations.

Surgical resection is the core treatment for malignant brain tumors [8, 21]. Consistent with existing literature, our data confirmed that surgical intervention inevitably leads to temporary declines in multiple HRQoL domains due to surgical stress and brain tissue irritation [22, 23]. Nevertheless, most functional indicators can gradually recover within one month, proving the overall safety and tolerance of craniotomy for MBT patients. Regular monitoring of patient-reported symptoms and timely supportive care are essential to improve perioperative experiences.

Tumor laterality was also found to correlate with a series of symptoms and functional domains. Left-hemisphere lesions were associated with more severe baseline communication impairment, while right-sided tumors linked to worse emotional, cognitive and social function in the early postoperative period. This phenomenon aligns with the well-documented functional lateralization of the human cerebrum [24, 25]. From a clinical perspective, recognizing hemispheric differences can help clinicians make individualized assessment plans and deliver targeted rehabilitation interventions for speech, cognition and motor function [24]. When interpreting findings related to tumor laterality and other associated factors, full caution is required. Our cohort consists of clinically heterogeneous glioma and BM patients. In addition, we performed a large number of statistical tests across multiple HRQoL domains and follow-up time points, which elevated the risk of Type I error and potential false-positive results. Tumor location, pathological grade and surgical extent also acted as confounding variables. Therefore, the observed associations are population-specific and cannot be generalized to all MBT patients. Further large-sample, stratified analyses are needed for external validation. Moreover, most patients with brain metastases received various systemic anti-tumor treatments prior to intracranial surgery. Such prior therapies are associated with patients’ baseline physical and psychological status as well as perioperative HRQoL changes. Since we did not stratify analyses by pre-treatment history, relevant associations could not be fully isolated in the current study.

Patient performance status (KPS) is closely correlated with pain, fatigue and other core symptoms, serving as an important reference for clinical decision-making [10, 12]. Compared with glioma patients, those with brain metastases presented worse symptom scores at baseline. This is largely because most BM patients had received systematic treatment for primary tumors before neurosurgery, resulting in accumulated symptom burden [7, 8]. Stratified analysis based on tumor grade also revealed obvious differences in baseline HRQoL between LGG and HGG, further indicating that tumor pathological characteristics are key factors affecting patients’ self-reported outcomes [15].

This study has several limitations. A notable drawback is the high follow-up attrition rate. We adopted multiple imputation to manage missing data, but sensitivity analysis was not performed due to small overall and subgroup samples. Limited sample size and the single-center design also restricted long-term follow-up and extended analysis. The 3-month follow-up window was relatively short, and the long-term association between HRQoL and overall survival remains to be explored. Attrition bias was unavoidable because patients in poorer general condition were more likely to be lost to follow-up [26]. Baseline KPS data were incomplete for patients with brain metastases due to insufficient medical records. In addition, we failed to collect detailed information on pre-operative systemic therapies and postoperative adjuvant treatments for MBT patients. These unrecorded treatment-related factors introduced residual confounding effects. Extensive multiple comparisons across domains, time points and subgroups also increased the risk of false-positive findings. In future work, we will recruit larger cohorts from multiple centers, collect complete pre- and post-treatment data, and conduct sensitivity and stratified analyses to minimize various biases.

In conclusion, MBT patients commonly experience transient declines in HRQoL in the early postoperative stage, with most indicators recovering within one month. Age, tumor laterality, surgical approach and KPS are independently associated with different HRQoL domains across follow-up time points. Our findings provide practical references for individualized follow-up, supportive treatment and perioperative management. Comprehensive assessment of HRQoL throughout the treatment course helps clinicians identify patients’ actual needs, optimize supportive care strategies, and ultimately improve the overall treatment experience and quality of life for patients with malignant brain tumors [11].

## Supplementary Information

Below is the link to the electronic supplementary material.

ESM 1 (DOCX 21.2 KB)

ESM 2 (DOCX 18.3 KB)

## Data Availability

No datasets were generated or analysed during the current study.

## Abbreviations

MBT: Malignant Brain Tumors
BM: Brain Metastases
GBM: Glioblastoma multiforme
HRQoL: Health-related Quality of Life
GHS: Global Health Status
PF: Physical Function
RF: Role Function
EF: Emotional Function
CF: Cognitive Function
SF: Social Function
FA: Fatigue
NV: Nausea and Vomiting
PA: Pain
DY: Dyspnea
IN: Insomnia
AL: Appetite Loss
CO: Constipation
DI: Diarrhea
FD: Financial Difficulties
FU: Future Uncertainty
VD: Visual Disturbance
MD: Motor Dysfunction
CD: Communication Deficit
HA: Headache
SE: Seizures
DR: Drowsiness
HL: Hair Loss
IS: Itching Skin
WL: Weakness of the Legs
BC: Bladder Control
MLR: Multivariate linear regression
