# Supplementary material for: Health-related quality of life and its determinants in patients with malignant brain tumors: a prospective longitudinal study
Source: Support Care Cancer. 2026 Sep 26;34(10):1021. doi: 10.1007/s00520-026-11262-0 (PMC13615918; doi:10.1007/s00520-026-11262-0)
Supplement: Supplementary file 1 [file 520_2026_11262_MOESM1_ESM.docx]

**Supplementary Table 1. Results of the linear mixed model analyses of the one month follow-up showing the association between several independent variables and the selected HRQoL scales (Gliomas).**

| **Variable** | **PF**  Beta (95% CI) | **NV**  Beta (95% CI) | **AL**  Beta (95% CI) | **FU**  Beta (95% CI) | **MD**  Beta (95% CI) | **CD**  Beta (95% CI) | **WL**  Beta (95% CI) |
| --- | --- | --- | --- | --- | --- | --- | --- |
| **Male** | Ref | Ref | Ref | Ref | Ref | Ref | Ref |
| **Female** | -9.9 (-24.3, 4.5) | 5.7 (-0.3, 11.6) | 6.0 (-11.1, 23.0) | -3.4 (-17.4, 10.6) | 6.8 (-5.5, 19.1) | -20.2 (-35.2, -5.2)* | -0.3 (-20.1, 19.4) |
| **Age, year** | -0.3 (-1.1, 0.5) | 0.1 (-0.2, 0.5) | 0.9 (-0.1, 1.8) | 0.3 (-0.5, 1.1) | 0.5 (-0.2, 1.2) | 0.4 (-0.4, 1.2) | 1.3 (0.2, 2.4)* |
| **BMI, kg/m2** | 0.7 (-1.7, 3.2) | -0.3 (-1.3, 0.8) | -1.1 (-4.0, 1.8) | -2.8 (-5.2, -0.4)* | -1.9 (-4.0, 0.2) | -3.7 (-6.3, -1.1)* | -4.2 (-7.5, -0.8)* |
| **Education, year** | -1.1 (-3.4, 1.2) | 0.01 (-1.0, 1.0) | 4.4 (1.6, 7.2)* | 0.8 (-1.5, 3.1) | 0.7 (-1.3, 2.7) | -0.3 (-2.7, 2.2) | 2.0 (-1.2, 5.2) |
| Biopsy only | Ref | Ref | Ref | Ref | Ref | Ref | Ref |
| Sub-resection | 23.9 (-9.5, 57.2) | 4.7 (-9.1, 18.5) | -5.1 (-44.7, 34.5) | -39.0 (-71.5, -6.5)* | -29.4 (-57.9, -0.9)* | -32.6 (-67.4, 2.3) | -24.2 (-70.0, 21.6) |
| Total-resection | 22.1 (0.6, 43.6)* | 4.2 (-4.7, 13.1) | 10.1 (-15.5, 35.7) | -17.8 (-38.7, 3.2) | -23.5 (-41.9, -5.2)* | -13.8 (-36.3, 8.7) | -23.8 (-53.3, 5.8) |
| **Grade** |  |  |  |  |  |  |  |
| I | Ref | Ref | Ref | Ref | Ref | Ref | Ref |
| II | -7.0 (-35.6, 21.5) | 1.0 (-10.8, 12.8) | 3.9 (-30.0, 37.8) | 17.5 (-10.3, 45.3) | -0.8 (-25.2, 23.6) | 23.5 (-6.3, 53.3) | 11.1 (-28.1, 50.3) |
| III | -14.7 (-44.1, 14.7) | 3.4 (-8.8, 15.6) | 4.6 (-30.3, 39.5) | 18.1 (-10.5, 46.8) | 4.9 (-20.2, 30.0) | 23.9 (-6.8, 54.6) | 18.4 (-22.0, 58.8) |
| IV | -12.5 (-43.2, 18.3) | 7.2 (-5.5, 19.9) | 16.7 (-19.8, 53.2) | 8.0 (-21.9, 37.9) | 5.9 (-20.4, 32.1) | 13.7 (-18.4, 45.8) | 15.7 (-26.5, 58.0) |
| **Side of the tumor** |  |  |  |  |  |  |  |
| Left hemisphere | Ref | Ref | Ref | Ref | Ref | Ref | Ref |
| Right hemisphere | 7.5 (-6.3, 21.3) | 5.8 (0.04, 11.5)* | 10.7 (-5.7, 27.1) | -3.9 (-25.2, 1.7) | -3.9 (-15.7, 7.9) | -40.3 (-54.7, -25.9)* | -9.4 (-28.4, 9.6) |
| Bilateral | - | - | - | - | - | - | - |
| **Tumor Location** |  |  |  |  |  |  |  |
| Frontal lobe | Ref | Ref | Ref | Ref | Ref | Ref | Ref |
| Temporal lobe | 0.6 (-16.6, 17.8) | 5.9 (-1.3, 13.0) | 12.0 (-8.4, 32.5) | 4.0 (-12.7, 20.8) | -1.0 (-15.7, 13.7) | 10.8 (-7.2, 28.7) | 0.2 (-23.4, 23.8) |
| Parietal lobe | 8.7 (-12.1, 29.6) | -6.9 (-15.5, 1.8) | 9.4 (-15.4, 34.1) | 1.5 (-18.8, 21.8) | -4.8 (-22.6, 13.0) | 14.4 (-7.4, 36.1) | 14.7 (-14.0, 43.3) |
| Occipital lobe | -25.4 (-72.6, 21.9) | 87.8 (68.3, 107.4)* | 89.6 (33.5, 145.8)* | -3.2 (-49.2, 42.9) | 44.9 (4.6, 85.3)* | -30.2 (-79.6, 19.1) | 55.6 (-9.4, 120.5) |
| Insula lobes | - | - | - | - | - | - | - |
| Multiple Lobes | 25.9 (3.3, 48.5)* | -5.9 (-15.2, 3.5) | -8.7 (-35.5, 18.2) | -16.9 (-38.9, 5.2) | -27.3 (-46.6, -8.0)* | -20.9 (-44.5, 2.7) | -28.2 (-59.3, 2.8) |
| Others | 22.1 (-17.6, 61.7) | -8.5 (-24.9, 8.0) | -10.1 (-57.2, 37.1) | -13.7 (-52.3, 25.0) | -16.5 (-50.4, 17.4) | 4.4 (-37.0, 45.8) | -17.2 (-71.7, 37.3) |
| Newly diagnosis | Ref | Ref | Ref | Ref | Ref | Ref | Ref |
| Recurrence | 6.3 ( -11.9, 24.6) | 8.2 (0.6, 15.7)* | 8.5 (-13.3, 30.2) | 5.9 (-11.9, 23.7) | -4.1 (-19.7, 11.5) | 1.6 (-17.5, 20.7) | -9.3 (-34.4, 15.8) |
| KPS | 0.4 (-0.3, 1.1) | -0.1 (-0.4, 0.2) | 0.2 (-0.7, 1.0) | -0.2 (-0.9, 0.5) | -0.04 (-0.6, 0.6) | 0.2 (-0.5, 1.0) | 0.5 (-0.4, 1.5) |

*P < 0.05; Ref, reference; Preop, Preoperation; postop, postoperation;
