# Supplementary material for: Health-related quality of life and its determinants in patients with malignant brain tumors: a prospective longitudinal study
Source: Support Care Cancer. 2026 Sep 26;34(10):1021. doi: 10.1007/s00520-026-11262-0 (PMC13615918; doi:10.1007/s00520-026-11262-0)
Supplement: Supplementary file 2 [file 520_2026_11262_MOESM2_ESM.docx]

**Supplementary Table 2. Results of the linear mixed model analyses of the three months follow-up, showing the association between several independent variables and the selected HRQoL scales (gliomas).**

| **Variable** | **GHs**  Beta (95% CI) | **PF**  Beta (95% CI) | **RF**  Beta (95% CI) | **EF**  Beta (95% CI) | **CF**  Beta (95% CI) | **SF**  Beta (95% CI) | **FD**  Beta (95% CI) | **MD**  Beta (95% CI) |
| --- | --- | --- | --- | --- | --- | --- | --- | --- |
| **Male** | Ref | Ref | Ref | Ref | Ref | Ref | Ref | Ref |
| **Female** | 6.1 (-14.3, 26.5) | 9.6 (-6.8, 26.1) | 5.0 (-9.6, 19.6) | 10.2 (-5.0, 25.4) | 23.1 (1.1, 45.0)* | 21.5 (0.6, 42.5)* | -20.8 (-44.4, 2.7) | -3.5 (-24.3, 17.3) |
| **Age, year** | 0.4 (-0.6, 1.4) | -0.3 (-1.1, 0.6) | 0.1 (-0.6, 0.8) | 0.6 (-0.1, 1.4) | 0.1 (-1.0, 1.2) | 1.1 (0.04, 2.1)* | -2.1 (-3.2, -0.9)* | -0.1 (-1.2, 0.9) |
| **BMI, kg/m2** | 1.8 (-0.8, 4.4) | 1.0 (-1.1, 3.1) | 1.3 (-0.6, 3.1) | -0.1 (-2.0, 1.8) | 2.1 (-0.7, 4.9) | 0.8 (-1.9, 3.4) | -0.4 (-3.4, 2.5) | -1.1 (-3.7, 1.6) |
| **Education, year** | -1.2 (-5.5, 3.2) | -2.4 (-5.9, 1.2) | -2.7 (-5.8, 0.5) | -2.9 (-6.1, 0.4) | 2.6 (-2.1, 7.3) | -2.4 (-6.9, 2.1) | -3.9 (-9.0, 1.1) | 2.2 (-2.3, 6.6) |
| Biopsy only | Ref | Ref | Ref | Ref | Ref | Ref | Ref | Ref |
| Sub-resection | 23.4 (-24.6, 71.3) | 18.4 (-20.3, 57.1) | 12.7 (-21.6, 46.9) | -2.8 (-38.5, 32.9) | 72.3 (20.7, 123.8) | 46.0 (-3.3, 95.2) | -30.7 (-86.0, 24.7) | -14.5 (-63.4, 34.3) |
| Total-resection | 10.7 (-25.6, 47.0) | 4.3 (-25.1, 33.6) | 29.6 (3.7, 55.6)* | 6.9 (-20.1, 34.0) | 24.6 (-14.4, 63.7) | 44.6 (7.3, 81.9)* | -40.7 (-82.7, 1.2) | -10.0 (-46.9, 27.1) |
| **Grade** |  |  |  |  |  |  |  |  |
| I | Ref | Ref | Ref | Ref | Ref | Ref | Ref | Ref |
| II | -23.8 (-75.6, 28.0) | -16.6 (-58.4, 25.3) | -11.5 (-48.5, 25.6) | 5.7 (-32.9, 44.2) | -46.3 (-102.0, 9.4) | -29.5 (-82.8, 23.7) | 14.4 (-45.4, 74.2) | 5.8 (-47.0, 58.5) |
| III | -39.2 (-91.0, 12.6) | -14.4 (-56.2, 27.4) | -11.3 (-48.3, 25.7) | 5.5 (-33.0, 44.0) | -35.1 (-90.8, 20.5) | -29.7 (-82.9, 23.4) | 20.7 (-39.1, 80.4) | 8.6 (-44.1, 61.3) |
| IV | -19.7 (-71.9, 32.6) | -22.4 (-64.6, 19.8) | -12.7 (-50.0, 24.7) | 6.6 (-32.3, 45.5) | -64.2 (-120.4, -8.1)* | -22.9 (-76.6, 30.8) | 54.7 (-5.6, 115.0) | 15.0 (-38.2, 68.2) |
| **Side of the tumor** |  |  |  |  |  |  |  |  |
| Left hemisphere | Ref | Ref | Ref | Ref | Ref | Ref | Ref | Ref |
| Right hemisphere | 2.0 (-17.9, 22.0) | 13.1 (-3.0, 29.3) | 7.5 (-6.8, 21.8) | 0.8 (-14.0, 15.7) | 34.3 (12.9, 55.8)* | 18.0 (-2.5, 38.5) | -0.8 (-23.9, 22.2) | -8.4 (-28.7, 11.9) |
| Bilateral | - | - | - | - | - | - | - | - |
| **Tumor Location** |  |  |  |  |  |  |  |  |
| Frontal lobe | Ref | Ref | Ref | Ref | Ref | Ref | Ref | Ref |
| Temporal lobe | 2.3 (-24.1, 28.7) | -0.7 (-22.0, 20.6) | 0.9 (-17.9, 19.8) | 18.7 (-0.9, 38.4) | -22.2 (-50.6, 6.2) | -7.8 (-34.9, 19.3) | -11.5 (-42.0, 18.9) | -12.7 (-39.6, 14.1) |
| Parietal lobe | 27.6 (-0.9, 56.0) | 4.3 (-18.7, 27.2) | 10.5 (-9.8, 30.8) | 17.8 (-3.3, 39.0) | -21.2 (-51.8, 9.3) | 14.8 (-14.4, 44.0) | 36.7 (3.9, 69.5)* | -18.1 (-47.0, 10.8) |
| Occipital lobe | 4.3 (-52.2, 60.8) | 16.9 (-28.7, 62.6) | 24.1 (-16.3, 64.5) | 14.9 (-27.2, 56.9) | 50.3 (-10.4, 111.0) | 18.6 (-39.4, 76.7) | -20.0 (-85.1, 45.3) | -38.9 (-96.4, 18.6) |
| Insula lobes | - | - | - | - | - | - | - | - |
| Multiple Lobes | 24.2 (-11.6, 60.0) | 18.4 (-10.5, 47.3) | 39.6 (14.0, 65.2)* | 17.0 (-9.6, 43.6) | 27.2 (-11.2, 65.7) | 37.2 (0.4, 73.9)* | -21.4 (-62.7, 19.9) | -25.0 (-61.4, 11.5) |
| Others | - | - | - | - | - | - | - | - |
| Newly diagnosis | Ref | Ref | Ref | Ref | Ref | Ref | Ref | Ref |
| Recurrence | -6.9 (-30.2, 16.5) | 5.5 (-13.4, 24.4) | -1.2 (-17.9, 15.5) | -5.3 (-22.7, 12.1) | -12.3 (-37.4, 12.8) | 3.8 (-20.2, 27.8) | -26.7 (-53.7, 0.2) | 4.0 (-19.8, 27.8) |
| KPS | 1.0 (0.04, 1.9) | 1.5 (0.7, 2.3)* | 1.7 (1.1, 2.4)* | 1.5 (0.8, 2.2)* | 0.4 (-0.6, 1.5) | 1.7 (0.7, 2.7)* | -0.7 (-1.8, 0.4) | -1.5 (-2.5, -0.6)* |

*P < 0.05; Ref, reference; Preop, Preoperation; postop, postoperation;
